# Altered fibroblast-like synoviocyte epigenetics is responsible for deficient NUB1 expression in rheumatoid arthritis

**DOI:** 10.1038/s41598-026-38420-y

**Published:** 2026-02-10

**Authors:** Yosuke Ono, Camilla R. L. Machado, Eunice Choi, David L. Boyle, Wei Wang, Gary S. Firestein

**Affiliations:** 1 Division of Rheumatology, Autoimmunity and Inflammation, San Diego School of Medicine, University of California, La Jolla, CA 92093 USA; 2 Department of Chemistry and Biochemistry, University of California, San Diego, La Jolla, CA 92093 USA

**Keywords:** Rheumatoid arthritis, Synovium, Fibroblast, Epigenetics, Neddylation, Diseases, Immunology, Molecular biology, Rheumatology

## Abstract

Neddylation is a post-translational modification suppressed by the endogenous inhibitor NUB1, and its dysregulation in rheumatoid arthritis (RA) promotes inflammation and NF-κB activation. NUB1 expression in RA and osteoarthritis (OA) synovium and mechanisms underlying defective NUB1 induction in RA fibroblast-like synoviocytes (FLS) were evaluated. NEDD8 and IL-6 protein expression was increased and NUB1 was reduced in RA synovium compared with OA tissue, with significant differences in the lining region that correlated with higher cytokine expression and NF-kB translocation. IL-1β–induced NUB1 induction was impaired in RA FLS at both the mRNA and protein levels. To evaluate the mechanism, we assessed mRNA stability using actinomycin D, examined the role of SNHG12 by siRNA knockdown, analyzed MAP kinase signaling, and measured NUB1 promoter activity with a luciferase reporter assay. None could explain the reduced induction observed in RA FLS. Treatment with the DNA methylation inhibitor 5-azacytidine and the histone methylation inhibitor EPZ6438 partially reversed the difference in NUB1 induction, whereas the histone deacetylase inhibitors ITF2375 and MS275 eliminated it. Therefore, defective NUB1 induction in RA FLS is related to the aberrant epigenetic landscape in RA FLS and is associated with increased neddylation and increased IL-6 expression in rheumatoid synovium. Overcoming increased neddylation in RA represents a novel therapeutic approach.

**Supplementary Information:**

The online version contains supplementary material available at 10.1038/s41598-026-38420-y.

## Introduction

Rheumatoid arthritis (RA) is a systemic immune-mediated disease characterized by chronic synovitis and progressive joint destruction^1^. Although recent therapeutic advances have improved the treatment of RA, a substantial proportion of patients fail to achieve adequate disease control or sustained remission^2^. Most current therapeutics act by suppressing immune cell function, which can also compromise host defense^3,4^. Identifying novel targets that can potentially regulate inflammation and tissue destruction without relying on immune suppression remains an unmet clinical need. Fibroblast-like synoviocytes (FLS) would represent a target that spares adaptive immunity in RA. These cells exhibit enhanced production of inflammatory cytokines such as IL-6, increased proliferation and invasion, and persistent in situ activation in RA, all of which contribute to the maintenance of inflammation^5,6^. However, the molecular mechanisms that establish and sustain the persistent inflammatory FLS phenotype are not completely understood^7^.

Neddylation is a post-translational modification pathway in which the ubiquitin-like molecule neuronal precursor cell-expressed developmentally down-regulated protein 8 (NEDD8) is covalently conjugated to cullin-RING E3 ubiquitin ligase (CRL) complexes, particularly the Skp1–Cullin1–F-box (CUL1; SCF)^8,9^. This modification enhances the E3 ligase activity of CRLs, thereby promoting the ubiquitination of their substrate proteins and accelerating proteasome-dependent degradation. Previously we showed that RA FLS have deficient NUB1 induction after IL-1 stimulation. In RA FLS, this leads to excessive neddylation, which accelerates IκB degradation (a downstream targeted transcription factor in this pathway)and in turn drives NF-κB nuclear translocation and IL-6 production^10^. These findings suggest that impaired NUB1 induction in RA amplifies inflammatory signaling and might serve as a potential therapeutic target. However, it remains unclear whether the molecular events observed in vitro,namely reduced NUB1 expression, accumulation of NEDD8, enhanced NF-κB activation, and increased IL-6 expression,are reflected in rheumatoid the synovial tissue. In addition, the mechanisms responsible for the impaired NUB1 induction in RA FLS remain unresolved.

In this study, we compared the expression levels and spatial localization of NUB1, NEDD8, and IL-6 as well as NF-κB activation in synovial tissues from RA and OA patients. We then evaluated the mechanisms that contribute to deficient NUB1 induction in RA FLS compared with osteoarthritis (OA) FLS. Through these tissue- and cell-based approaches, we provide evidence that epigenetic dysregulation in RA plays a central role, raising the possibility remodeling these epigenetic abnormalities might help restore homeostasis.

## Results

### Reduced NUB1 expression correlates with high NEDD8 and IL-6 protein expression in RA synovium

**Fig. 1:**
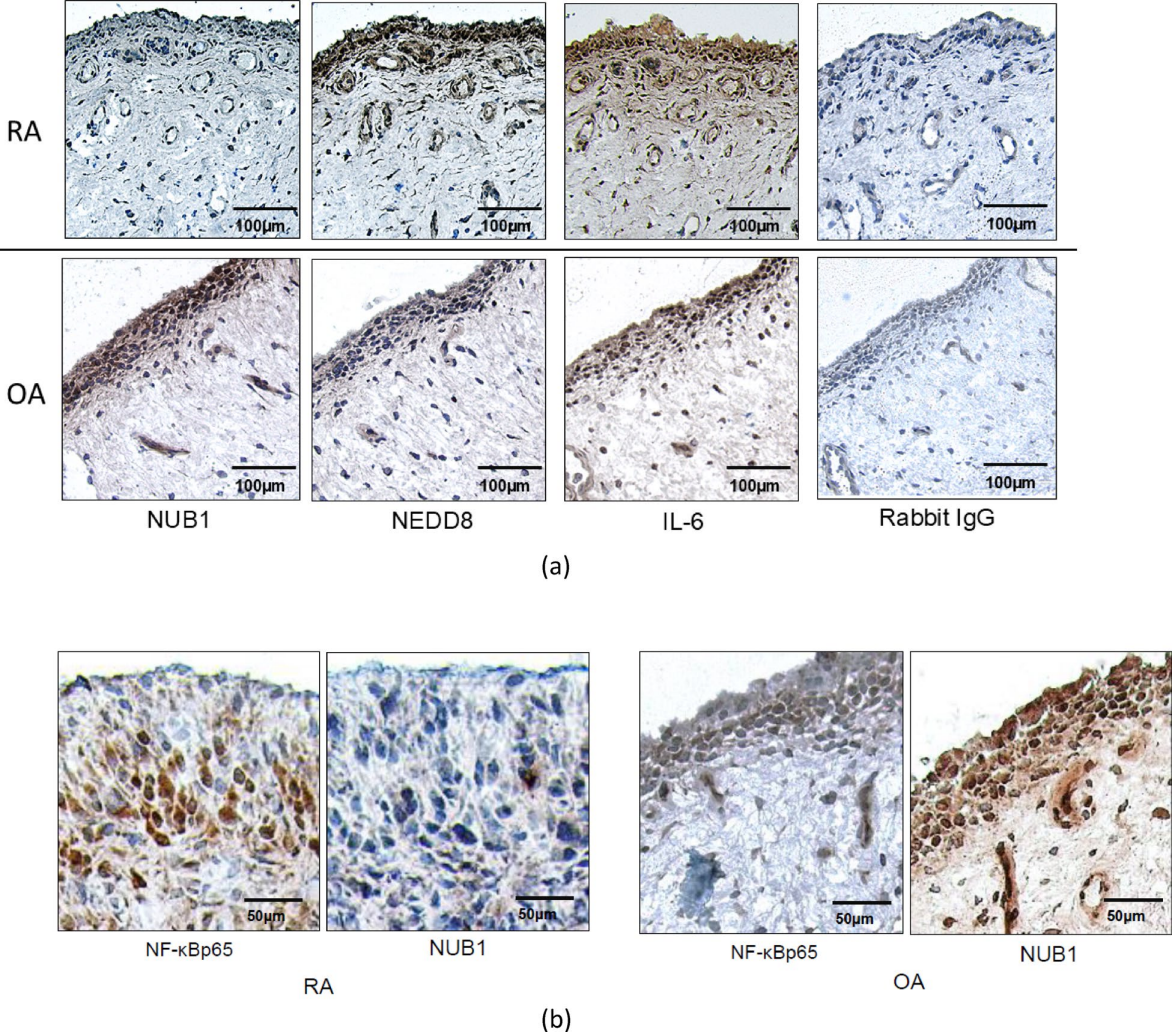
Immunohistochemical staining of NUB1, NEDD8, IL-6, NF-kB in RA and OA synovial tissues. (**a**) Representative images (magnification, ×200) of immunohistochemistry for NUB1, NEDD8, and IL-6 in synovial tissues from patients with RA and OA. Expression of NEDD8 and IL-6 was higher in the intimal lining of RA synovium, and expression of NUB1 was lower compared with OA (see Fig. 2 for quantification). Scale bar = 100 μm, (**b**) Representative images (magnification, ×400) of immunohistochemistry for NUB1 and p65 in synovial tissues from patients with RA and OA. In RA, regions with reduced NUB1 expression showed prominent nuclear localization of p65, whereas in OA, areas with higher NUB1 expression displayed weaker p65 nuclear staining. Negative control (NC) sections were stained with rabbit IgG under identical conditions. Scale bar = 50 μm.

We previously demonstrated that IL-1–induced NUB1 expression is attenuated in RA FLS compared with OA FLS^10^. To determine whether this differential induction is also evident in synovial tissue, we performed immunohistochemistry to assess relative expression of NUB1 in synovial tissues. Representative images are provided in Fig. 1a and show that NUB1 protein primarily localized in the synovial intimal lining where FLS reside. After confirming and localizing NUB1 expression, we then addressed two questions, namely whether NUB1 protein expression was higher in OA than RA tissue, and whether low NUB1 expression is associated with high NEDD8 and IL-6 expression and nuclear localization of p65, a subunit of the NF-κB transcription factor complex.

A semiquantitative scoring system was used to determine relative protein expression levels of NUB1, NEDD8, and IL-6 in synovial tissues (*n* = 5 per group). We predicted that low NUB1 expression in RA would correlate with higher expression of neddylation pathway–related markers, including NEDD8, as well as increased IL-6 expression.

**Fig. 2:**
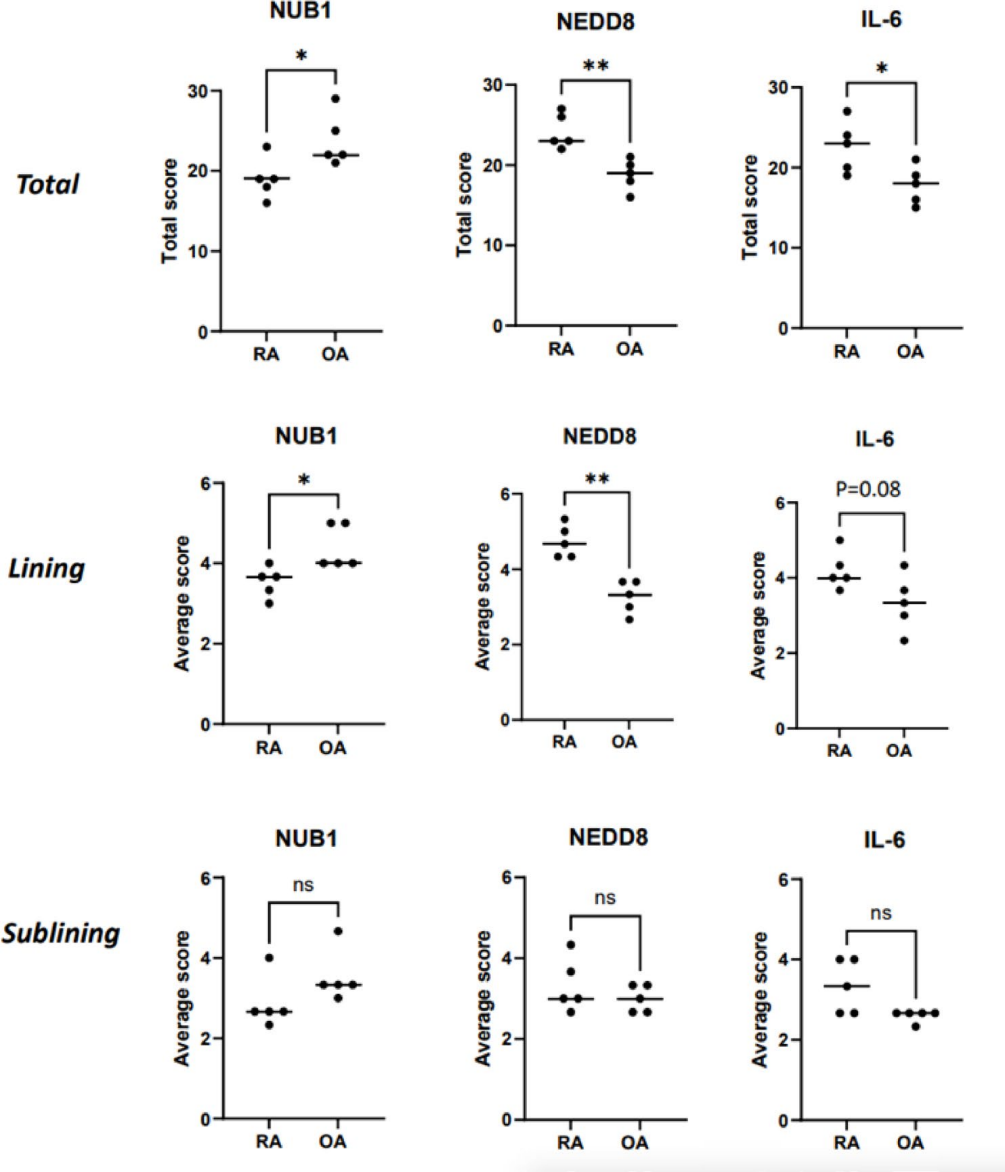
Immunohistochemical scoring of NUB1, NEDD8, and IL-6 in RA and OA synovial tissues. Synovial tissue sections from RA (n = 5) and OA (n = 5) patients were immunostained for NUB1, NEDD8, IL-6, and CCL5. For each patient, three randomly selected fields were evaluated in the lining and sublining zones. Staining was semiquantitatively scored as described in Methods. Graphs show the total scores as well as the average scores for the lining and sublining zones. For the total scores, NUB1 was higher in OA than in RA, whereas NEDD8 and IL-6 were significantly higher in RA; these differences were most prominent in the lining layer. Data are presented as mean ± SEM. Statistical analysis was performed using unpaired *t* test with Welch’s correction or Mann-Whitney *U* test (*p<0.05; **p<0.01).

As shown in Fig. 2, this was indeed the case: total scores revealed that NUB1 expression was significantly lower in RA (*p* = 0.043), and NEDD8 and IL-6 expression were significantly higher in RA (NEDD8; *p* = 0.010, IL-6; *p* = 0.026). Much of this difference was accounted for by the lining, where the score of NUB1 was lower in RA (*p* = 0.032), while that of NEDD8 was higher in RA than OA (*p* = 0.008), along with a trend towards higher levels of IL6 (*p* = 0.08). In addition, we examined the expression of p65, a subunit of the NF-κB transcription factor complex, in relation to NUB1 (Fig. 1b). p65 nuclear was prominent in areas with low NUB1 expression. In contrast, in OA, regions with high NUB1 expression showed only weak nuclear staining of p65. These findings are consistent with the fact that NUB1 inhibits NF-κB activation by decreasing IκB degradation^10^. Overall, these data show an association between the expression of NUB1 and the relative expression of downstream genes that are regulated by this pathway.

### Defective IL-1-induced NUB1 expression in RA FLS 

**Fig. 3:**
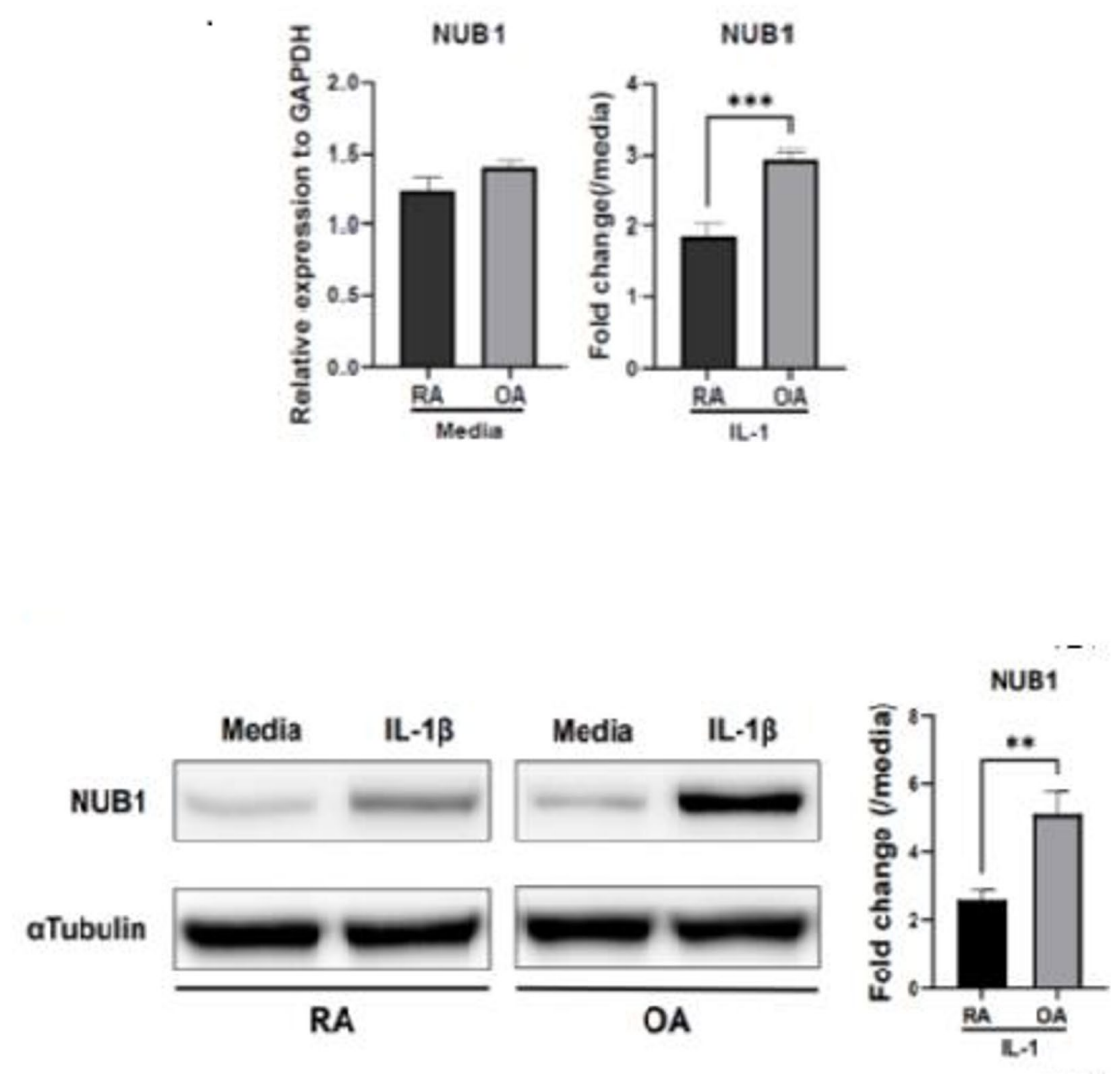
Basal and IL-1-induced expression of NUB1 in RA and OA FLS. (**a**) Left panel: Basal NUB1 mRNA levels in RA and OA FLS were quantified by RT-qPCR and normalized to GAPDH. Right panel: Following stimulation with IL-1β (2 ng/mL) for 6 h, the fold change in NUB1 expression relative to unstimulated cells was calculated for each group (n = 6 each). Basal NUB1 mRNA expression was comparable between RA and OA FLS (*p* = 0.153), whereas IL-1β–induced upregulation of NUB1 was significantly lower in RA FLS than in OA FLS. (**b**) NUB1 protein expression in RA and OA FLS following IL-1β stimulation. Left panel: RA and OA FLS were stimulated with IL-1β (2 ng/mL) for 24 h, and NUB1 protein expression was evaluated by Western blotting. Right panel: NUB1 band intensities were quantified after normalization to α-tubulin. IL-1β-induced NUB1 protein expression was also significantly reduced in RA FLS compared with OA FLS. These indicate defective IL-1–mediated NUB1 induction in RA FLS. Data are presented as mean ± SEM. Statistical significance was determined using unpaired *t* test with Welch’s correction. (**p < 0.01, ***p < 0.001).

To understand the mechanisms underlying the induction of NUB1 in RA FLS, we first compared its basal and IL-1–stimulated expression between RA and OA FLS. At baseline, NUB1 mRNA levels were comparable in RA and OA FLS. However, following IL-1β stimulation, the induction of NUB1 was significantly attenuated in RA FLS compared with OA FLS at both the mRNA and protein levels (Fig. 3a and b, Supplementary Figs. 1 and 2). These findings confirm our previous observations^10^. Subsequent studies focused on dissecting the potential mechanisms that might underlie low NUB1 induction in RA FLS.

### NUB1 induction by IL-1 is not dependent on MAP kinases 

**Fig. 4:**
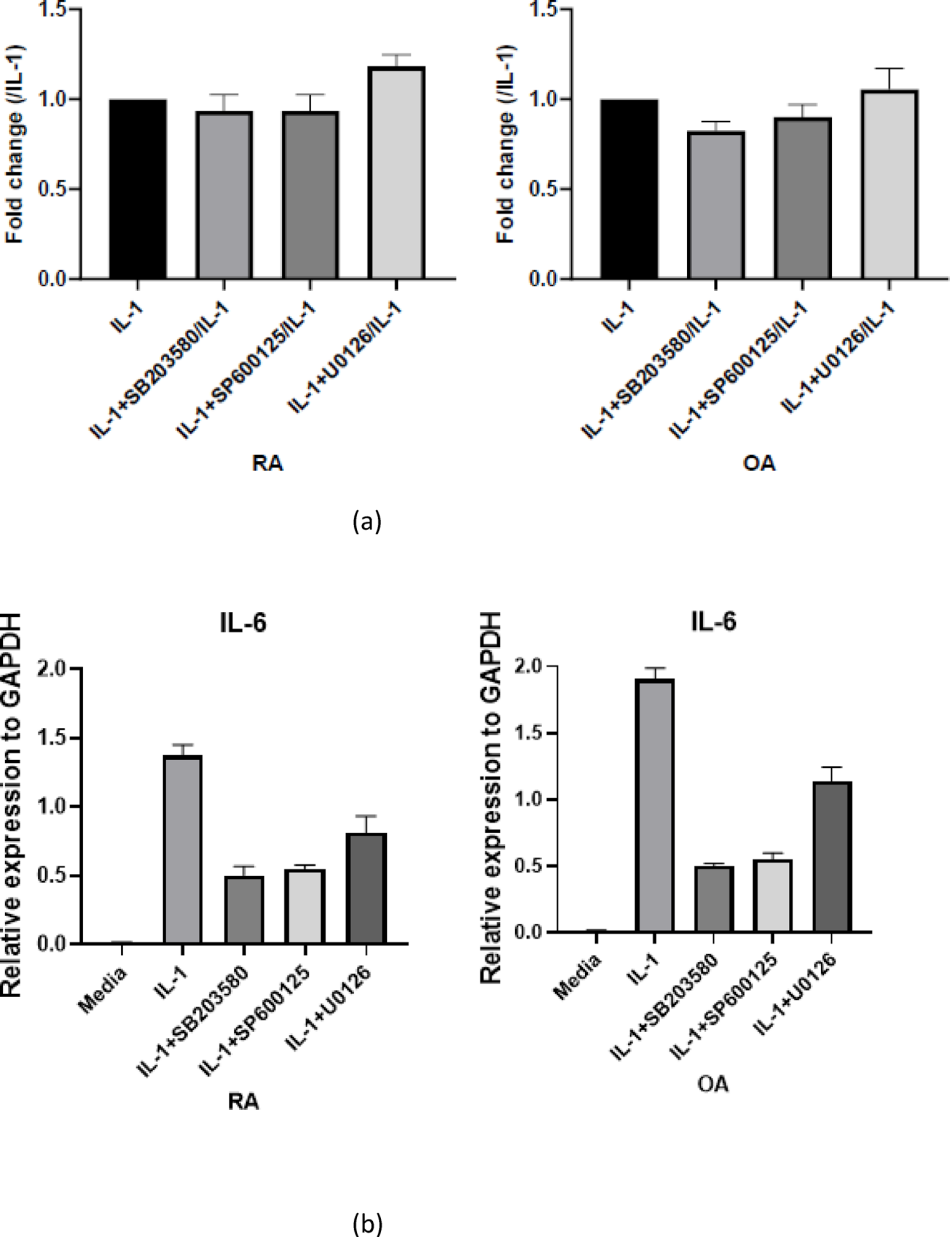
Regulation of NUB1 expression by MAPKs . RA and OA FLS (n = 5 each) were stimulated with IL-1β (2 ng/mL) for 6h in the presence or absence of MAPK inhibitors: SP600125 (JNK inhibitor), SB203580 (p38 MAPK inhibitor), and U0126 (MEK1/2–ERK inhibitor). NUB1 mRNA expression is shown as fold change relative to IL-1β stimulation alone. (**a**) Quantitative analysis demonstrated that none of the MAPK inhibitors significantly reduced IL-1β–induced NUB1 mRNA expression in either RA or OA FLS (IL-1β vs. IL-1β + SB203580, adjusted *p* > 0.9999; IL-1β vs. IL-1β + SP600125, adjusted *p* > 0.9999; IL-1β vs. IL-1β + U0126, adjusted *p* = 0.8285). (**b**) IL-6 mRNA expression was quantified by RT-qPCR and normalized to GAPDH. IL-6 expression was significantly reduced by each MAPK inhibitor compared with IL-1β stimulation alone (****p < 0.0001), confirming effective inhibition of MAPK-dependent signaling. Data are presented as mean ± SEM. Statistical significance was determined using one-way ANOVA with Tukey’s multiple comparisons test.

After confirming that IL-1β stimulation recapitulates the defective induction of NUB1 in RA FLS compared with OA, we next sought to determine whether the differential induction of NUB1 by IL-1β was due to aberrant upstream signaling, we first analyzed the effects of MAP kinase pathway inhibitors. When RA and OA FLS were stimulated with IL-1β in the presence of SP600125 (JNK inhibitor), SB203580 (p38 inhibitor), or U0126 (MEK1/2–ERK inhibitor), showed that none of the MAPK inhibitors significantly reduced IL-1β–induced NUB1 mRNA expression in either group (Fig. 4a). Supplementary Fig. 3 shows the deficient induction of NUB1 in this experiment.

In contrast, the positive control gene IL-6 showed a significant reduction in mRNA levels (Fig. 4b), confirming effective inhibition of MAPK-dependent signaling. Together, these results indicate that MAPK signaling is unlikely to be a determinant of IL-1β–induced NUB1 expression, and is unlikely to explain the impaired NUB1 induction observed in RA. This led us to investigate other downstream regulatory mechanisms.

### Mechanisms of defective IL-1-induced NUB1 expression

#### mRNA stability

**Fig. 5:**
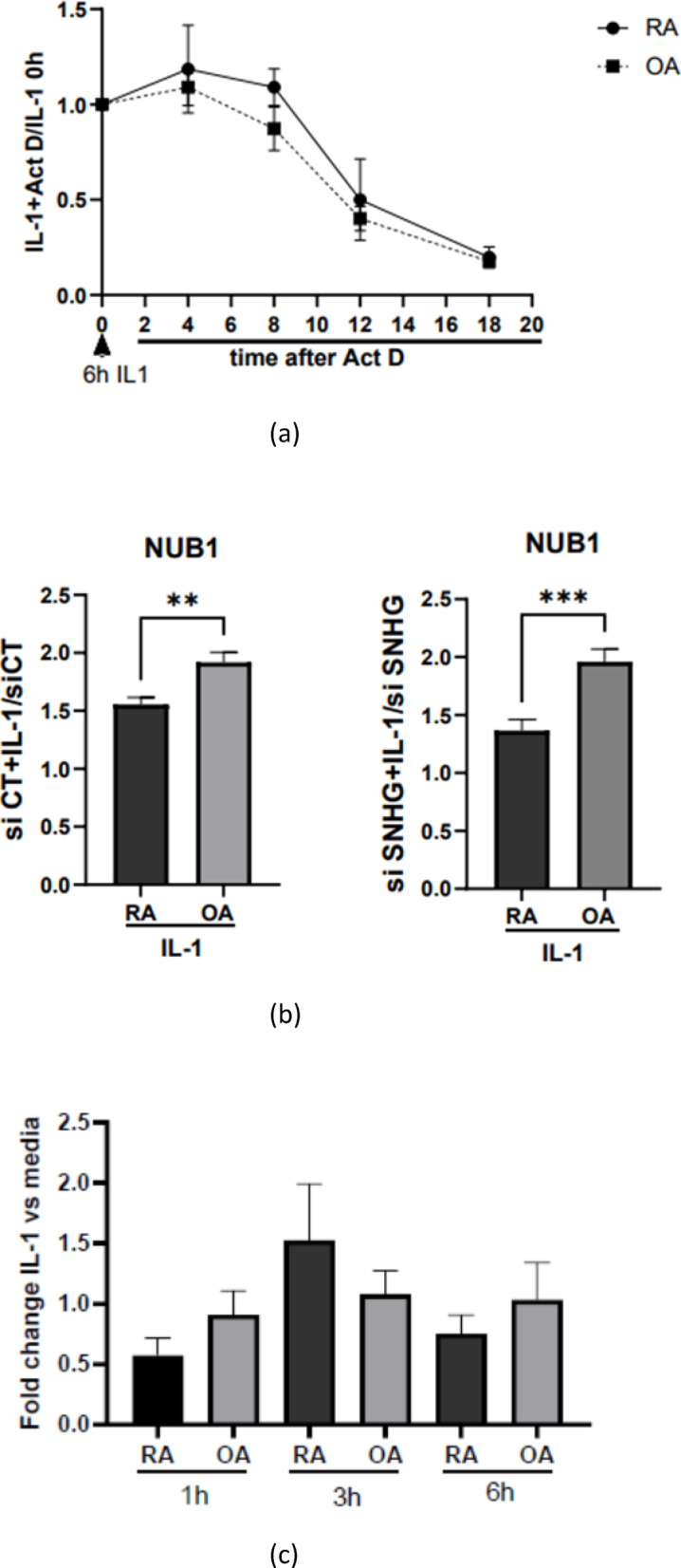
(**a**) mRNA half life of NUB1. RA and OA FLS (n = 5 each) were stimulated with IL-1β (2 ng/mL) for 6 h, followed by treatment with actinomycin D (10 μg/mL) to block transcription. NUB1 mRNA expression was then measured by qRT-PCR at 0, 4, 8, 12, and 18 h after actinomycin D treatment. Expression levels were normalized to GAPDH and presented as fold change relative to the 0 h time point (i.e., the level observed immediately after 6 h of IL-1β stimulation). Decay curves showed no significant differences between groups. mRNA decay curves were fitted using a one-phase exponential decay model by nonlinear regression to calculate NUB1 mRNA half-life, and decay constants (K) for RA and OA were compared using an extra sum-of-squares *F*-test. (**b**) Effect of SNHG12 knockdown on NUB1 expression in RA and OA FLS. SNHG12 (Small Nucleolar RNA Host Gene 12), a long non-coding RNA associated with NUB1, was depleted in RA and OA FLS using SNHG12 siRNA (knockdown efficiency >80%). RA and OA (n = 7 each) FLS were stimulated with IL-1β (2 ng/mL) for 6 h, and NUB1 mRNA expression was quantified by RT-qPCR and normalized to GAPDH. Left panel: Fold change in NUB1 expression following IL-1β stimulation in siCT-transfected cells (IL-1/unstimulated). Right panel: Fold change in NUB1 expression following IL-1β stimulation after SNHG12 knockdown (siSNHG12 + IL-1/siSNHG12 unstimulated). Knockdown of SNHG12 had little effect on the RA–OA difference in NUB1 induction. Data are presented as mean ± SEM. Statistical analysis was performed using unpaired *t* test with Welch’s correction (**p < 0.01, ***p < 0.001). (**c**) NUB1 promoter activity in RA and OA FLS. RA and OA FLS (n = 5 each) were transfected with a luciferase reporter construct containing the NUB1 promoter region and co-transfected with Renilla vector for normalization (see Material and Methods). After transfection, cells were stimulated with IL-1β (2 ng/mL). Firefly luciferase activity was normalized to Renilla and expressed as fold change calculated by dividing the IL-1β–stimulated value by the corresponding unstimulated (media) control at each time point. Limited promoter induction was noted, possibly because transcription requires epigenetic marks or the three-dimensional structure of chromatin not present in plasmids. No significant differences in IL-1β–induced NUB1 promoter activity were observed between RA and OA FLS at 1 h (*p* = 0.209), 3 h (*p* = 0.415), or 6 h (*p* = 0.446). Data are presented as mean ± SEM. Statistical analysis was performed using an unpaired *t* test with Welch’s correction.

To explore the molecular mechanisms underlying the reduced induction of NUB1 in RA compared with OA, we then examined the potential contribution of post-transcriptional regulation. RA and OA FLS were stimulated with IL-1β for 6 h, followed by transcriptional blockade with actinomycin D. NUB1 mRNA decay was monitored over time using qPCR. Expression levels at each time point were normalized to the baseline. This analysis assessed post-induction mRNA decay kinetics rather than the amplitude of transcriptional induction (see Fig. 5a). The decay kinetics were comparable between RA and OA FLS, with calculated half-lives of 11.3 h and 10.7 h, respectively, indicating that differences in mRNA stability are unlikely to explain the reduced induction in RA (Fig. 5a). GAPDH expression remained stable throughout the actinomycin D treatment period indicating that there was not significant cell death (Supplementary Fig. 4).

#### Small nucleolar RNA host gene 12

We next examined whether Small Nucleolar RNA Host Gene 12 (SNHG12) contributes to differential NUB1 induction. SNHG12 is a long non-coding RNA previously reported to regulate NUB1 expression^11^.

We first assessed endogenous SNHG12 expression in RA and OA FLS under the same experimental conditions used for NUB1 analyses. SNHG12 mRNA levels were comparable between RA and OA FLS at baseline and after IL-1β stimulation (Supplementary Fig. 5). To determine whether SNHG12 contributes to the defective IL-1β–induced transcriptional upregulation of NUB1 in RA FLS, we depleted this lncRNA using siRNA (Supplementary Fig. 6). Despite SNHG12 knockdown, IL-1β–induced NUB1 mRNA expression remained significantly lower in RA FLS than in OA FLS, and the differences in NUB1 induction between RA and OA were not reversed (Fig. 5b). Consistent with this finding, within-group analyses in RA FLS demonstrated that although IL-1β stimulation significantly increased NUB1 mRNA expression under both control and SNHG12 knockdown conditions, SNHG12 depletion did not augment the magnitude of IL-1β–induced NUB1 expression or reverse the defect in RA FLS (Supplementary Fig. 7). These results indicate that differential SNHG12 expression or function does not explain impaired IL-1β–mediated transcriptional induction of NUB1 observed in RA FLS.

#### Promoter function

 We also investigated whether intrinsic promoter activity contributed to reduced NUB1 induction observed in RA. RA and OA FLS were transfected with a luciferase reporter vector containing the NUB1 promoter region and then stimulated with IL-1β. Promoter-driven luciferase activity was quantified as fold change relative to the corresponding unstimulated (medium) control at each time point. Interestingly, the isolated promoter had limited activity as measured by luciferase even when cells were stimulated with IL-1β. In other experiments, we also showed that the promoter could be modestly activated by other stimuli, such as IFNβ (Supplementary Fig. 8).

As a control, we observed that IL-1β increased activation of an NF-κB–responsive luciferase reporter in both RA and OA FLS (Supplementary Fig. 9), confirming that IL-1/NF-κB signaling was effectively engaged in this experimental system. However, the magnitude and kinetics of IL-1β fold induction were comparable between groups, with no significant differences detected at 1 h, 3 h, or 6 h (Fig. 5c). These findings indicate that IL-1β–dependent transcriptional regulation of NUB1 is not detected within this isolated proximal promoter fragment. While promoter involvement cannot be completely excluded, the reduced NUB1 induction observed in RA is unlikely to be explained by differences in intrinsic activity of this proximal promoter region alone.

### Epigenetic modulators reverse the differential induction of NUB1 

After confirming that attenuated induction of NUB1 in RA is not due to differences in mRNA stability, regulation by SNHG12, MAP kinase signaling, or intrinsic promoter activity, we evaluated the role of differential epigenetic marks in FLS. Previous studies of epigenetic marks in and around the NUB1 gene using ChIPseq revealed enriched marks for H3K27ac and mark H3K27me3 in OA compared with in RA. These data suggested that the promoter is poised in OA but not in RA as a possible mechanism. We tested this possibility by altering epigenetic marks of FLS.

#### DNA methylation

**Fig. 6:**
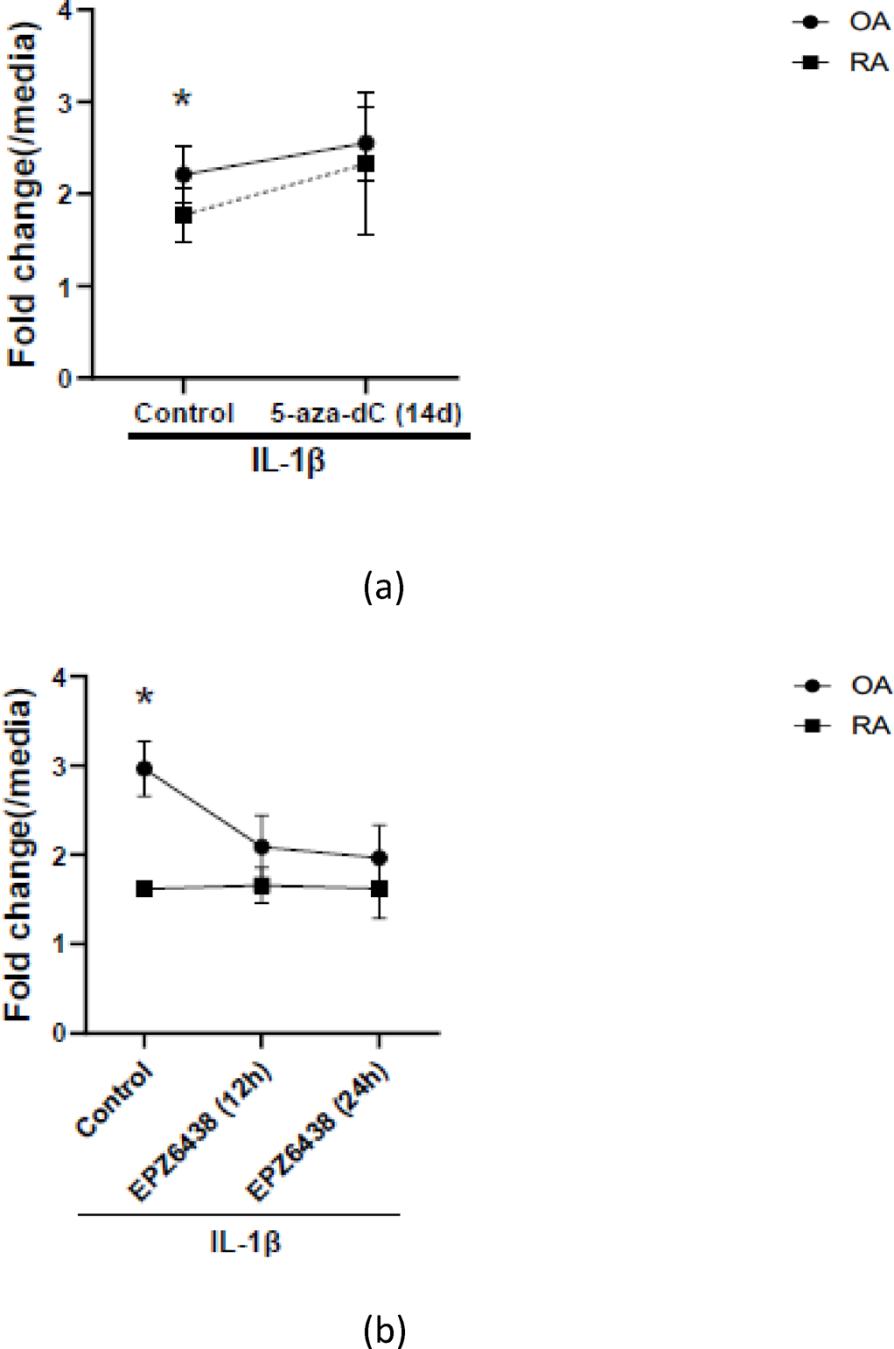

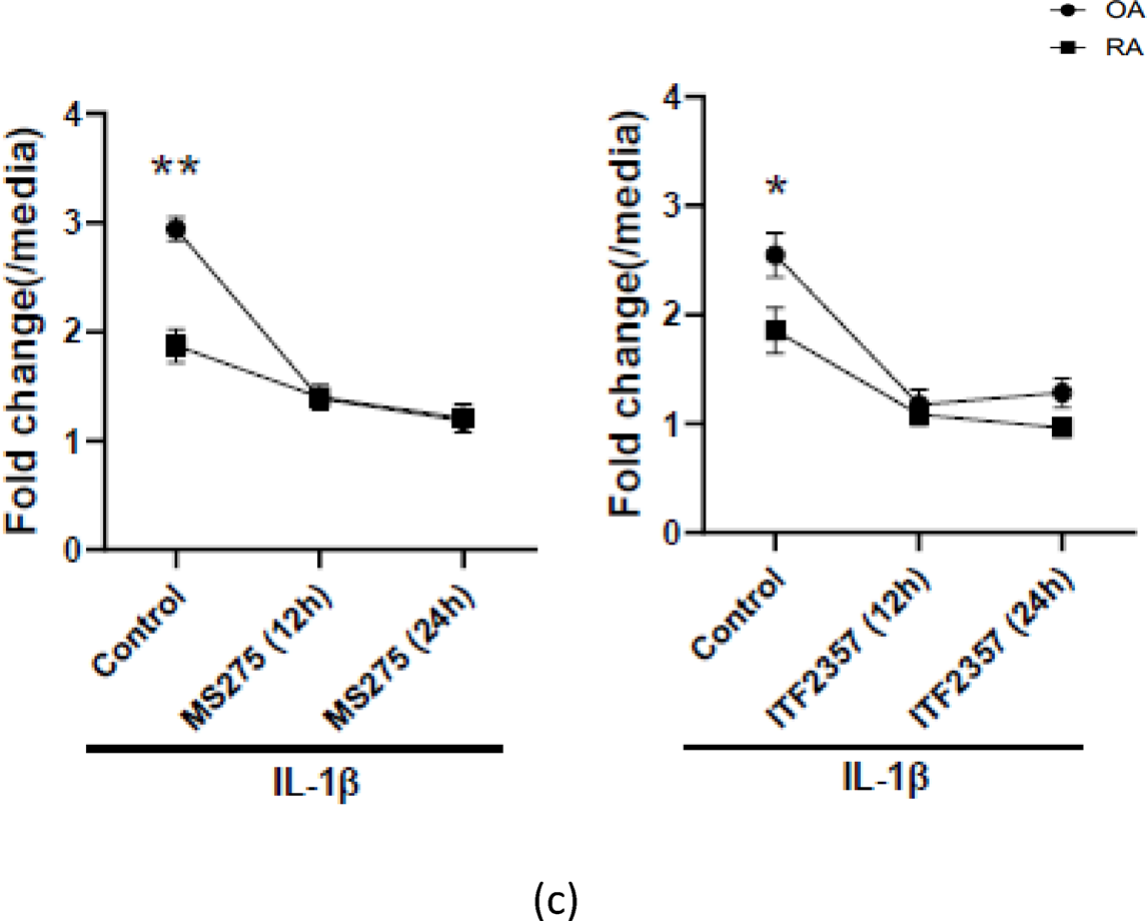
Effects of epigenetic inhibitors on IL-1β–induced NUB1 expression in RA and OA fibroblast-like synoviocytes (FLS). RA and OA FLS were pretreated with epigenetic inhibitors and subsequently stimulated with IL-1β (2 ng/mL). NUB1 mRNA expression was quantified by qRT-PCR. Fold change was calculated as the ratio of NUB1 mRNA expression in IL-1β–stimulated cells to the corresponding unstimulated control; specifically, DMSO alone for the IL-1/DMSO condition and the inhibitor-alone control for inhibitor-treated conditions. (**a**) 5-aza-deoxycytidine (5-aza-dC; DNA methyltransferase inhibitor). RA and OA FLS were treated with 5-aza-dC for 14 days prior to stimulation with IL-1β (n = 7 each). 5-aza-dC partially reduced the difference in IL-1β–induced NUB1 expression between RA and OA. Under control conditions, IL-1β–induced NUB1 expression was significantly higher in OA than in RA (*p* = 0.024). Following 5-aza-dC treatment, the magnitude of the OA–RA difference was no longer statistically significant (*p* = 0.488), indicating partial attenuation of the baseline RA–OA difference. (**b**) EPZ6438 (EZH2 inhibitor; histone methylation inhibitor). RA and OA FLS were treated with EPZ6438 for 12 or 24 hours before IL-1β stimulation (n = 5 each). treatment with EPZ6438 partially reversed the difference in IL-1β–induced NUB1 expression between RA and OA. Under control conditions, IL-1β–induced NUB1 expression was significantly higher in OA than in RA. This difference was no longer significant after EPZ6438 treatment at 12 h (*p* = 0.329) or 24 h (*p* = 0.512). (**c**) Left panel: ITF2357 (pan-HDAC inhibitor); Right panel: MS275 (HDAC1/3 selective inhibitor). RA and OA FLS were treated with each HDAC inhibitor for 12 or 24 hours, followed by IL-1β stimulation (ITF2357; n = 5 each, MS275; n = 6 each). Under control conditions, IL-1β–induced NUB1 expression was significantly higher in OA than in RA in the ITF2357 panel. Following ITF2357 treatment, the OA–RA difference was no longer statistically significant at 12 h (*p* = 0.596) or 24 h (*p* = 0.072). Under control conditions, IL-1β–induced NUB1 expression was also significantly higher in OA than in RA in the MS275 panel. Following MS275 treatment, the OA–RA difference was no longer statistically significant at either 12 h (*p* = 0.944) or 24 h (*p* = 0.846). These data indicate that histone modifications are required for differential induction of NUB1 in OA compared with RA. Circles and squares represent mean fold change for OA and RA FLS, respectively. Statistical analysis was performed at each time point and condition performed by comparing the fold change values between RA and OA FLS using unpaired *t* test with Welch’s correction (*p < 0.05, **p < 0.01).

 We first examined the effect of generalized DNA demethylation in RA and OA FLS using 5-azadeoxycytidine (5-aza-dC), as previously described^12^. Under control (baseline) conditions, the IL-1β–induced fold change in NUB1 expression was significantly higher in OA FLS than in RA FLS. RA and OA FLS were then treated with 5-aza-dC for 2 weeks, followed by IL-1β stimulation, and the IL-1β–induced fold change in NUB1 expression was compared between RA and OA FLS. As shown in Fig. 6a, 5-aza-dC treatment attenuated the baseline OA–RA difference in IL-1β–induced NUB1 expression, indicating that DNA methylation could contribute to the differential regulation of NUB1 between RA and OA FLS. Under these conditions, IL-1–induced IL-6 expression was maintained despite prolonged treatment with 5-aza-dC (Supplementary Fig. 10).

#### Histone methylation

FLS were pretreated with the EZH2 inhibitor EPZ6438 to reduce H3K27me3, followed by IL-1β stimulation. Under control (baseline) conditions, the IL-1β–induced fold change in NUB1 expression was higher in OA FLS than in RA FLS. RA and OA FLS were pretreated with EPZ6438 for 12 h, and at this time point, the IL-1β–induced fold change in NUB1 expression was directly compared between RA and OA FLS. EPZ6438 pretreatment partially attenuated the baseline OA–RA difference in NUB1 induction at 12 h. Specifically, the IL-1β–induced fold change was 1.6-fold in RA versus 3.0-fold in OA under control conditions, and was reduced to 1.6-fold in RA versus 2.0-fold in OA after 12 h of EPZ6438 pretreatment (Fig. 6b).

#### Histone acetylation

Histone deacetylation was then blocked with either the pan-HDAC inhibitor ITF2357 or the selective HDAC1/3 inhibitor MS275. Under control (baseline) conditions, the IL-1β–induced fold change in NUB1 expression was significantly higher in OA FLS than in RA FLS. RA and OA FLS were pretreated with each HDAC inhibitor for 12–24 h, followed by IL-1β stimulation.   At each time point the IL-1β–induced fold change in NUB1 expression was directly compared between RA and OA FLS. These agents eliminated the differential induction of NUB1. With MS275, the fold change shifted from 1.9 (RA) versus 2.9 (OA) under control conditions to 1.4 versus 1.4, and with ITF2357, from 1.9 (RA) versus 2.5 (OA) to 1.1 versus 1.2, indicating that histone-modifying inhibitors eliminated the differences between OA and RA FLS (Fig. 6c). Other IL-1 responses still occurred in the presence of the HDAC inhibitor conditions (Supplementary Figs. 11a, 12a). Collectively, these findings indicate that histone acetylation is likely be involved in the differential regulation of IL-1–induced NUB1 expression between RA and OA synovial fibroblasts. GAPDH Ct values remained stable in the presence of each inhibitor (Supplementary Figs. 10–12b).

## Discussion

In this study, we investigated whether the impaired induction of NUB1 observed in RA primary FLS also occurs in situ within synovial tissues. We further analyzed the regulatory mechanisms that could explain this phenomenon using FLS in an in vitro system. We first demonstrated that NUB1 expression was reduced in RA synovial tissues, and that areas with lower NUB1 expression showed correspondingly higher levels of NEDD8 and IL-6. These findings suggest that the molecular mechanism identified in vitro, namely, reduced NUB1 induction leading to excessive neddylation, activation of the NF-κB pathway, and increased IL-6 production, likely occurs in intact synovium.

We then explored a variety of potential mechanisms to explain deficient induction of NUB1 in RA FLS. While the limited effects on mRNA stability and MAP kinase activation were expected, we were surprised that SNHG12 and promoter activation had no effect. Previous studies suggested that this lncRNA could act as a molecular sponge for NUB1 in other cell lineages ^11^. Although SNHG12 can regulate NUB1 at the protein level in other cell types, our findings indicate that SNHG12 does not contribute to the defective IL-1β–induced transcriptional regulation of NUB1 in RA FLS. However, this was not observed in FLS and illustrates the importance of studying relevant cell lineages. The relative lack of function for the NUB1 promoter after IL-1 stimulations suggests that epigenetic marks or 3-dimensional chromatin structure that are not features of plasmid constructs are required.

The key finding from this study was that differences between RA and OA FLS with regard to NUB1 induction could be eliminated compounds that remodeled histone marks. We have previously shown that these modifiers, especially HDAC inhibitors, increase H3K27ac levels in FLS^13^. In RA FLS, epigenetic abnormalities in RA FLS—including enhanced histone deacetylation, increased repressive histone modifications, altered DNA methylation patterns, and remodeled chromatin accessibility—have been widely reported^12,14^. Among these, the relationship between inducible gene expression and epigenetic dysregulation has been described for classical inflammatory genes such as IL-6 and CXCL12^14^. However, the impact of epigenetic mechanisms on the inducible genes involved in the ubiquitin–neddylation pathway was largely unexplored. Our study provides novel insights by demonstrating that the impaired induction of NUB1 might be linked to underlying epigenetic alterations in RA FLS.

Impaired induction or reduced expression of NUB1 has been implicated in diseases outside of RA^15^. Several studies have reported that diminished NUB1 expression is associated with enhanced tumor cell proliferation and poor clinical outcomes in multiple cancer types^16,17^. Given that NUB1 functions as a negative regulator of the neddylation pathway by promoting the degradation of NEDD8-modified substrates^18^, loss of NUB1 might facilitate pathological neddylation activity in those diseases. Indeed, increased activation of the neddylation pathway has been implicated in tumor growth. However, to date, no studies have functionally demonstrated that impaired NUB1 inducibility arises from underlying epigenetic dysregulation. In this regard, our findings show that reduced NUB1 induction in RA FLS might be linked to epigenetic alterations, and that this could contribute to increased activation of transcription factors like NF-κB. These insights might also offer a new perspective for understanding the mechanisms underlying NUB1 induction defects in diseases beyond RA.

The present study has several limitations. Although we demonstrated that impaired NUB1 induction in RA FLS is associated with epigenetic dysregulation, the specific epigenetic region within the NUB1 locus that might contribute to this defect has not been identified. While locus-level analyses such as ChIP-qPCR or DNA methylation assays could, in principle, provide additional mechanistic insight, our findings and our previous map of the epigenetic landscape suggest that the impaired inducibility of NUB1 in RA FLS reflects dysregulation in larger regions rather than individual loci^19^. Epigenetic regulation of gene expression can involve multiple upstream interactions—including distal enhancers, chromatin looping, and differences in the availability of transcriptional cofactors—beyond promoter-proximal elements^20–22^, making the precise localization of causal regulatory sites inherently challenging. Moreover, targeted assays limited to selected regions may not capture distributed chromatin changes across the NUB1 regulatory regions. A comprehensive assessment of locus-specific regulation would likely require genome-wide approaches, such as ChIP-seq. In addition, the epigenetic inhibitors used in this study have broad activity, and the normalization of NUB1 induction could partly reflect off-target effects^23,24^. Nevertheless, the consistent elimination or marked reduction of RA–OA differences across multiple classes of epigenetic inhibitors supports a functional link between epigenetic regulation and defective NUB1 inducibility in RA FLS. Further validation using selective approaches might be informative. This study also focused primarily on IL-1–mediated induction, and the extent to which our findings generalize to other stimuli remains to be clarified.

In conclusion, this study demonstrates that impaired NUB1 induction in RA operates at both synovial tissue and FLS. Rather than arising from isolated promoter or post-transcriptional defects, this defect is associated with upstream epigenetic dysregulation. Together, these findings support the involvement of the NUB1–neddylation–NF-κB–IL-6 axis as a contributing pathway to persistent inflammation in RA and point to therapeutic strategies that restore NUB1 induction by remodeling epigenetic abnormalities.

## Materials and methods

### Synovial tissue collection and human fibroblast-Like synoviocytes

This study was approved by the Institutional Review Board of University. California, San Diego, and written informed consent was obtained from all participants (IRB #14–0175). All procedures were performed in accordance with the relevant guidelines and regulations. Synovial tissues were obtained from patients with rheumatoid arthritis (RA) or osteoarthritis (OA) undergoing total joint replacement or synovectomy. RA was diagnosed according to the 2010 ACR/EULAR criteria^25^, and OA according to established 1991 criteria^26^. Synovial tissues were minced and enzymatically digested as previously described^27^, and the resulting cells were cultured in Dulbecco’s Modified Eagle Medium (DMEM; Life Technologies) supplemented with 10% heat-inactivated fetal bovine serum (FBS; Gemini Bio-Products), penicillin, streptomycin, gentamicin, and L-glutamine in a humidified 5% CO_2_ atmosphere. Cells were allowed to adhere overnight, after which non-adherent cells were removed. Adherent FLS were expanded, split at a 1:3 ratio when 70–80% confluent, and used between passages 4 and 7. For all experiments, multiple independent FLS lines derived from different RA or OA patients were used. The number of cell lines analyzed for each assay is indicated in the corresponding figure legends.

### Antibodies and reagents

For immunohistochemistry, anti-NEDD8 (19E3) rabbit mAb (#2754), and anti-p65 rabbit mAb (#8242), were purchased from Cell Signaling Technology, and a rabbit polyclonal anti-NUB1 antibody (#14343-1-AP), and anti-rabbit polyclonal anti-IL-6 antibody (#21865-1-AP) were purchased from Proteintech, and normal Rabbit IgG control (#AB-105-C) was purchased from R༆D). VECTASTAIN Elite ABC-HRP Kit, Peroxidase (Rabbit IgG) (PK-6101), and ImmPACT DAB Substrate Kit, Peroxidase (HRP) (SK-4105) were purchased from Vector Laboratories. For Western blotting, a rabbit monoclonal anti-NUB1 antibody (#14810) and anti-α-tubulin rabbit mAb (#2125) were obtained from Cell Signaling Technology. Recombinant human IL-1β (407615) and IFNβ(8499-IF) were purchased from R&D System. The MAPK inhibitors, SB203580 (p38 MAPK inhibitor; R&D Systems, #1202), SP600125 (JNK inhibitor; Tocris, #1496), and U0126 (MEK inhibitor; Tocris, #1144) were used in this study. For epigenetic modulation experiments, we used the EZH2 inhibitor EPZ6438 (Tazemetostat; Selleck Chemicals), the pan-HDAC inhibitor ITF-2357 (givinostat; Selleck Chemicals), and the selective HDAC1/3 inhibitor MS-275 (entinostat; Selleck Chemicals), as previously described in synovial fibroblast studies^13^. For HDAC inhibition, we used MS275 and ITF2357 under comparable exposure conditions, as their target engagement has been validated in our experimental system^27^. For EZH2 inhibition, we used the EPZ6438 (EZH2 inhibitor; histone methylation inhibitor), whose ability to reduce trimethylation of histone H3 at lysine 27 (H3K27me3) is well established. EPZ6438 decreases H3K27me3 levels across multiple cellular systems and in vivo models, confirming effective target engagement of EZH2 inhibition^13^. For global DNA demethylation, cells were treated with the DNA methyltransferase inhibitor 5-aza-2′-deoxycytidine (Sigma-Aldrich), following established protocols for 14 days culture in FLS^12^.

### Real-time quantitative polymerase chain reaction

Total RNA was isolated from synovial tissues and FLS using the RNeasy Mini Kit (Qiagen) according to the manufacturer’s instructions. Complementary DNA (cDNA) was synthesized from 250 to 500 ng of total RNA using TaqMan™ Reverse Transcription Reagents (Thermo Fisher Scientific). The resulting cDNA served as a template for amplification by qPCR using gene-specific primers for NUB1 (human, Hs00997386_m1), IL-6 (human, Hs00174131_m1), and SNHG12 (Hs00414754_m1), which were purchased from Sigma-Aldrich, on a StepOne™ Real-Time PCR System (Thermo Fisher Scientific). Glyceraldehyde 3-phosphate dehydrogenase (GAPDH) was used as an internal control, and relative gene expression levels were quantified after normalization to GAPDH expression. For validation of RT-qPCR normalization, GAPDH Ct values were examined across all experimental conditions, including long-term epigenetic inhibitor treatments and actinomycin D time-course experiments.

### Western blot analysis

FLS were stimulated with IL-1β (2 ng/mL) for 24 h unless otherwise indicated. Western blot analysis was performed as previously described^28^. Cells were lysed with RIPA buffer supplemented with protease and phosphatase inhibitors. Protein concentration was measured using the Micro BCA™ Protein Assay Kit (Thermo Fisher Scientific), and 25 µg protein were separated by SDS-PAGE and transferred to PVDF membranes. Membranes were blocked with 5% milk in TBST, incubated with primary antibodies against NUB1 and αTubulin at 4℃. Horseradish peroxidase (HRP)-conjugated goat anti-rabbit IgG was used as secondary antibody. The signal was developed using Immun-Star WesternC ECL substrate (Bio-Rad), captured with a VersaDoc imaging system (Bio-Rad), and analyzed using ImageJ software. Protein expression levels were normalized to α-tubulin.

### Immunohistochemistry of synovial tissue

Frozen synovial sections (5 μm) were fixed in cold acetone for 10 min and prepared as previously described^29^. Endogenous peroxidase activity was blocked for 10 min using a dual endogenous enzyme block (Dako) according to the manufacturer’s instructions. After blocking with normal goat serum for 20 min, sections were incubated overnight at 4 °C with primary antibodies against NUB1 (1:400), NEDD8 (1:400), IL-6 (1:200), or p65 NF-κB (1:1500). Negative controls were stained with isotype-matched rabbit IgG. After washing, the slides were incubated with biotinylated secondary anti-rabbit antibodies for 30 min, treated with VECTASTAIN Elite ABC reagent (Vector Laboratories) for 30 min, and developed using ImmPACT DAB Substrate Kit (Vector Laboratories). Counterstaining was performed with Mayer’s hematoxylin. Images were acquired at ×200 or ×400 magnification using a bright-field microscope.

### Semi-quantitative immunohistochemistry scoring

Synovial tissue sections from RA (*n* = 5) and OA (*n* = 5) patients were immunostained for NUB1, NEDD8, IL-6, and p65. Semi-quantitative scoring was performed for NUB1, NEDD8, and IL-6 by a reader masked to the disease. The scoring framework was adapted from Krenn et al.^30^. For each patient, three randomly selected fields were evaluated separately in the intimal lining and sublining zones. In each field, staining was scored semi-quantitatively according to two parameters: (A) the proportion of positively stained cells (0–3) and (B) the staining intensity (0–3). These two scores were then summed to generate a composite score for that field, yielding a total score ranging from 0 to 6.

### mRNA stability assay

To assess NUB1 mRNA stability, RA and OA FLS (*n* = 5 each) were first stimulated with IL-1β (2 ng/mL) for 6 h and then treated with actinomycin D (10 µg/mL; #A9415, Sigma-Aldrich) to block transcription for up to 18 h. Total RNA was harvested at 0, 4, 8, 12, and 18 h after the addition of actinomycin D, and NUB1 mRNA expression was quantified by qRT-PCR. Expression levels were normalized to GAPDH and expressed as fold change relative to the 0 h time point (immediately after 6 h of IL-1β stimulation). This experimental design evaluates post-induction mRNA decay kinetics and does not assess the magnitude of the initial transcriptional induction. mRNA decay curves were fitted using nonlinear regression to calculate NUB1 mRNA half-lives.

### NUB1 promoter luciferase reporter assay

A luciferase reporter vector containing the human NUB1 promoter region (1241 bp) cloned into pGL4.23 (Promega) was used. The inserted fragment spans approximately − 1180 to + 60 bp relative to the major transcription start site (TSS) of NUB1, based on the canonical transcript annotation, and encompasses the proximal promoter region without distal enhancer elements. The sequence of the cloned promoter fragment was confirmed prior to use. RA and OA FLS were transfected with 1 µg of the promoter construct together with 50 ng of a Renilla control vector using the Human Dermal Fibroblast Nucleofector Kit (Lonza), as previously described. After 24 h, cells were stimulated with IL-1β (2 ng/mL), lysed, and analyzed using the Dual-Luciferase Reporter Assay System (Promega). Firefly luciferase activity was normalized to Renilla activity, and fold induction was calculated relative to the corresponding unstimulated (media) control at each time point. As a positive control for IL-1β–responsive transcriptional activity, an NF-κB–responsive luciferase reporter construct (pGL4.32[luc2P/NF-κB-RE/Hygro], Promega) was used in parallel experiments, as previously described^10^.

### Gene silencing

5 × 10^5^ FLS were transfected with 1 µg of SNHG12 (small interfering Smart Pool On-Target RNA, Horizon Discovery) or non-targeting control pool siRNA (Horizon Discovery) using the normal human dermal fibroblast Nucleofactor kit (Lonza), according to the manufacturer’s instruction^29^. Silencing efficiencies of SNHG was 80%.

### Statistics

Statistical analysis was performed using two-way *t* test, Mann-Whitney *U* test, or one-way analysis of variance (ANOVA) followed by Tukey’s test for multiple comparisons. For mRNA half-life analysis, decay curves were fitted using a one-phase exponential decay model. Differences in decay constants (K) between RA and OA were evaluated using an extra sum-of-squares *F*-test. All statistical analysis were performed using GraphPad Prism software (9.4.1). Results are presented as the mean ± SEM. A comparison was considered significant if *p* value was 0.05 or less.

## Supplementary Information

Below is the link to the electronic supplementary material.

Supplementary Material 1

## Data Availability

The datasets used and/or analyzed during the current study are available from the corresponding author on reasonable request.
