# Supplementary material for: Altered fibroblast-like synoviocyte epigenetics is responsible for deficient NUB1 expression in rheumatoid arthritis
Source: Sci Rep. 2026 Feb 10;16:8128. doi: 10.1038/s41598-026-38420-y (PMC12960655; doi:10.1038/s41598-026-38420-y)
Supplement: Supplementary file 1 — Supplementary Material 1 [file 41598_2026_38420_MOESM1_ESM.pptx]

## Slide 1
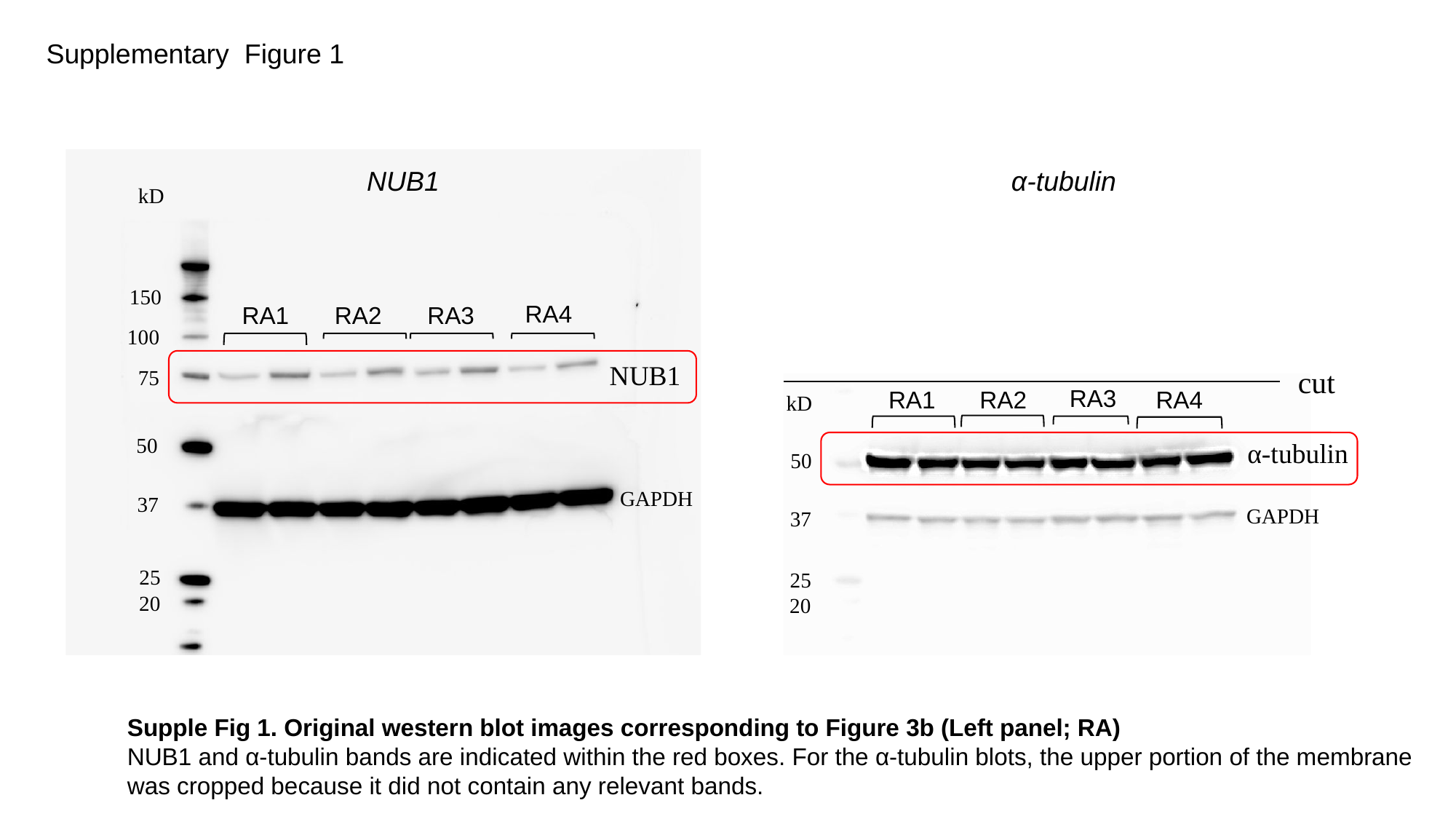

Supplementary Figure 1
NUB1
α-tubulin
kD
150
RA4
RA1
RA2
RA3
100
NUB1
cut
75
RA3
RA2
RA4
RA1
kD
50
α-tubulin
50
GAPDH
37
GAPDH
37
25
25
20
20
Supple Fig 1. Original western blot images corresponding to Figure 3b (Left panel; RA)
NUB1 and α-tubulin bands are indicated within the red boxes. For the α-tubulin blots, the upper portion of the membrane was cropped because it did not contain any relevant bands.

## Slide 2
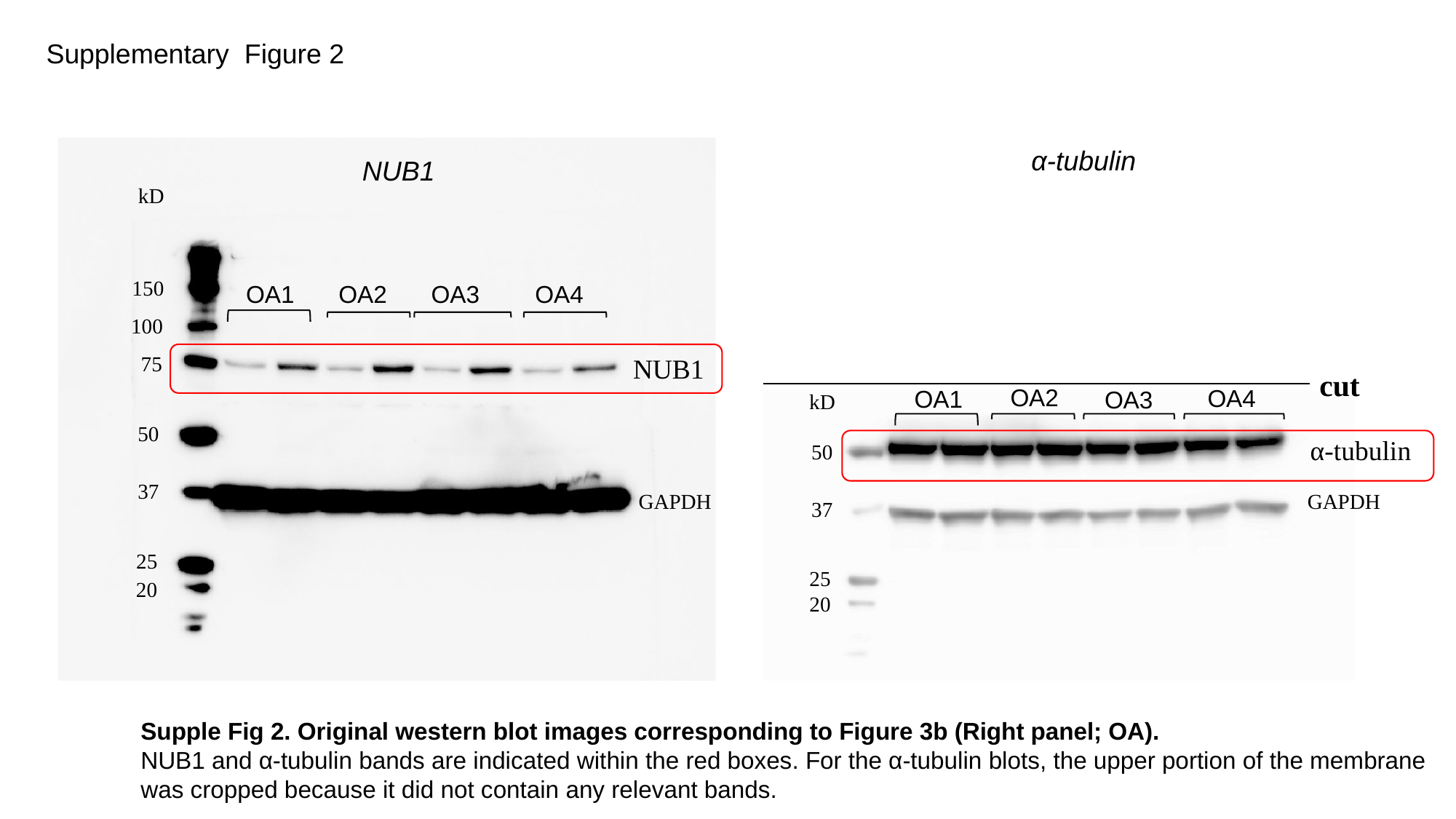

Supplementary Figure 2
α-tubulin
NUB1
kD
150
OA1
OA2
OA3
OA4
100
75
NUB1
cut
OA2
OA4
OA1
OA3
kD
50
α-tubulin
50
37
GAPDH
GAPDH
37
25
25
20
20
Supple Fig 2. Original western blot images corresponding to Figure 3b (Right panel; OA).
NUB1 and α-tubulin bands are indicated within the red boxes. For the α-tubulin blots, the upper portion of the membrane was cropped because it did not contain any relevant bands.

## Slide 3
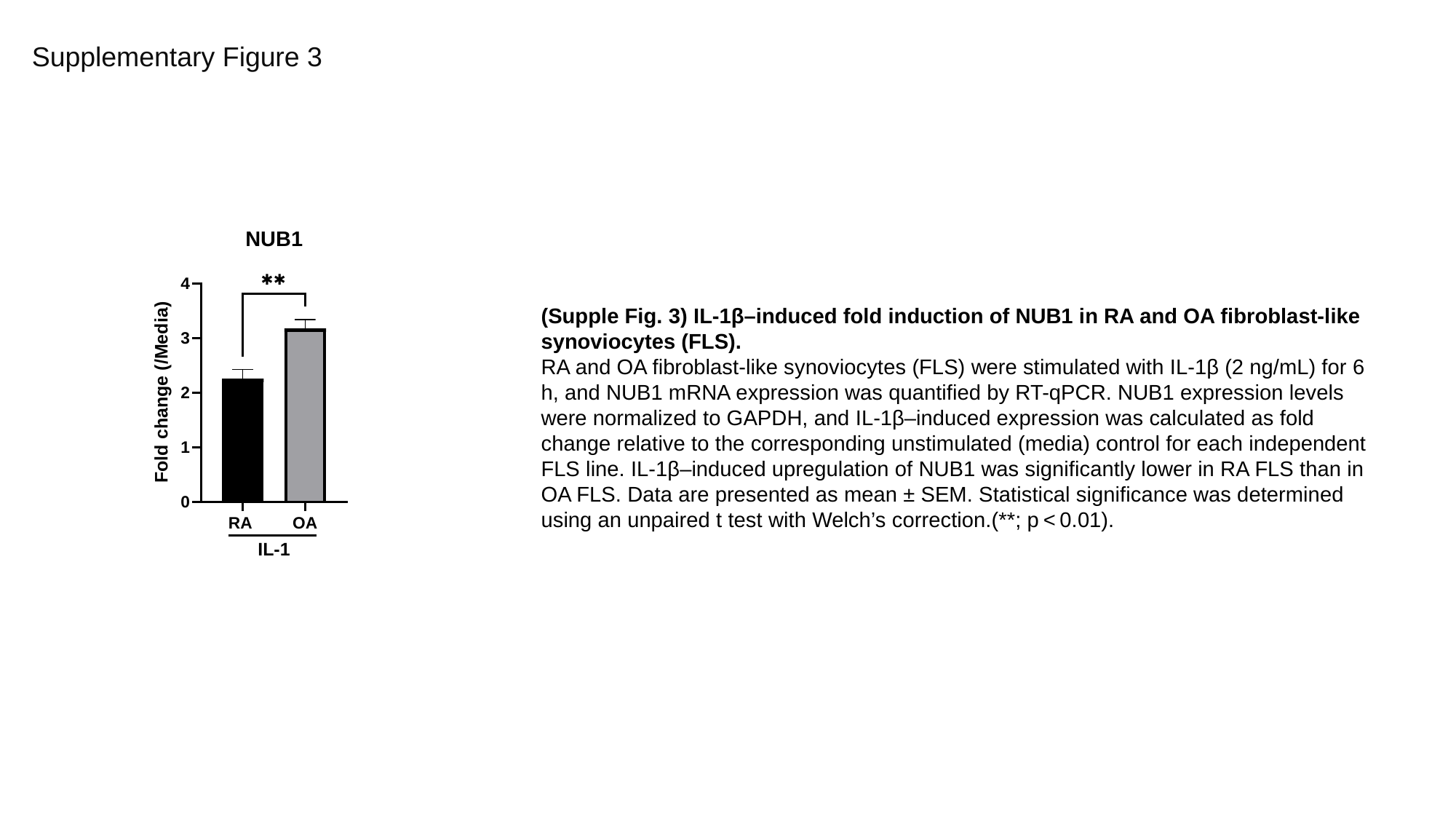

Supplementary Figure 3
(Supple Fig. 3) IL-1β–induced fold induction of NUB1 in RA and OA fibroblast-like synoviocytes (FLS).
RA and OA fibroblast-like synoviocytes (FLS) were stimulated with IL-1β (2 ng/mL) for 6 h, and NUB1 mRNA expression was quantified by RT-qPCR. NUB1 expression levels were normalized to GAPDH, and IL-1β–induced expression was calculated as fold change relative to the corresponding unstimulated (media) control for each independent FLS line. IL-1β–induced upregulation of NUB1 was significantly lower in RA FLS than in OA FLS. Data are presented as mean ± SEM. Statistical significance was determined using an unpaired t test with Welch’s correction.(**; p < 0.01).

## Slide 4
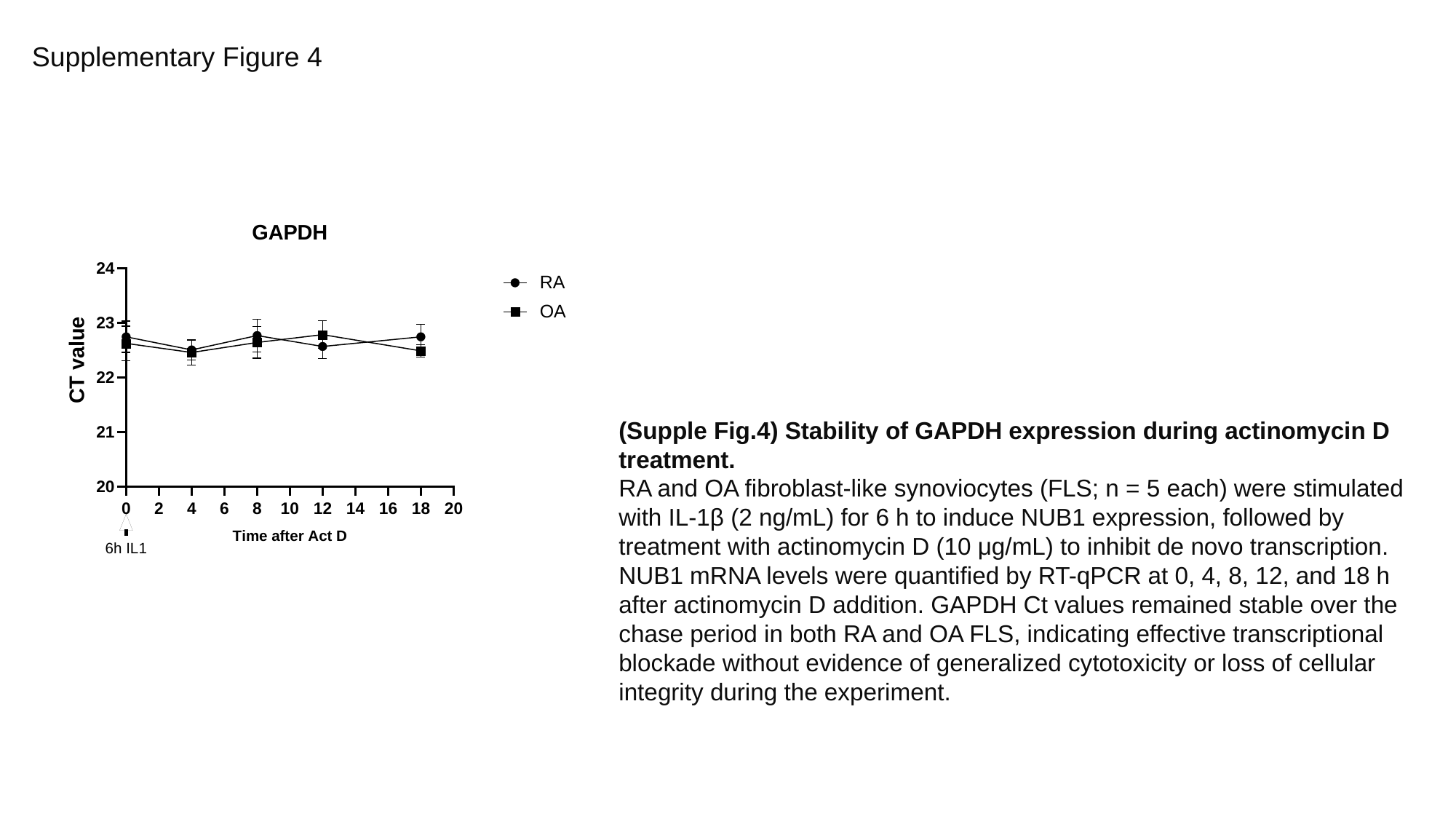

Supplementary Figure 4
(Supple Fig.4) Stability of GAPDH expression during actinomycin D treatment.
RA and OA fibroblast-like synoviocytes (FLS; n = 5 each) were stimulated with IL-1β (2 ng/mL) for 6 h to induce NUB1 expression, followed by treatment with actinomycin D (10 μg/mL) to inhibit de novo transcription. NUB1 mRNA levels were quantified by RT-qPCR at 0, 4, 8, 12, and 18 h after actinomycin D addition. GAPDH Ct values remained stable over the chase period in both RA and OA FLS, indicating effective transcriptional blockade without evidence of generalized cytotoxicity or loss of cellular integrity during the experiment.

## Slide 5
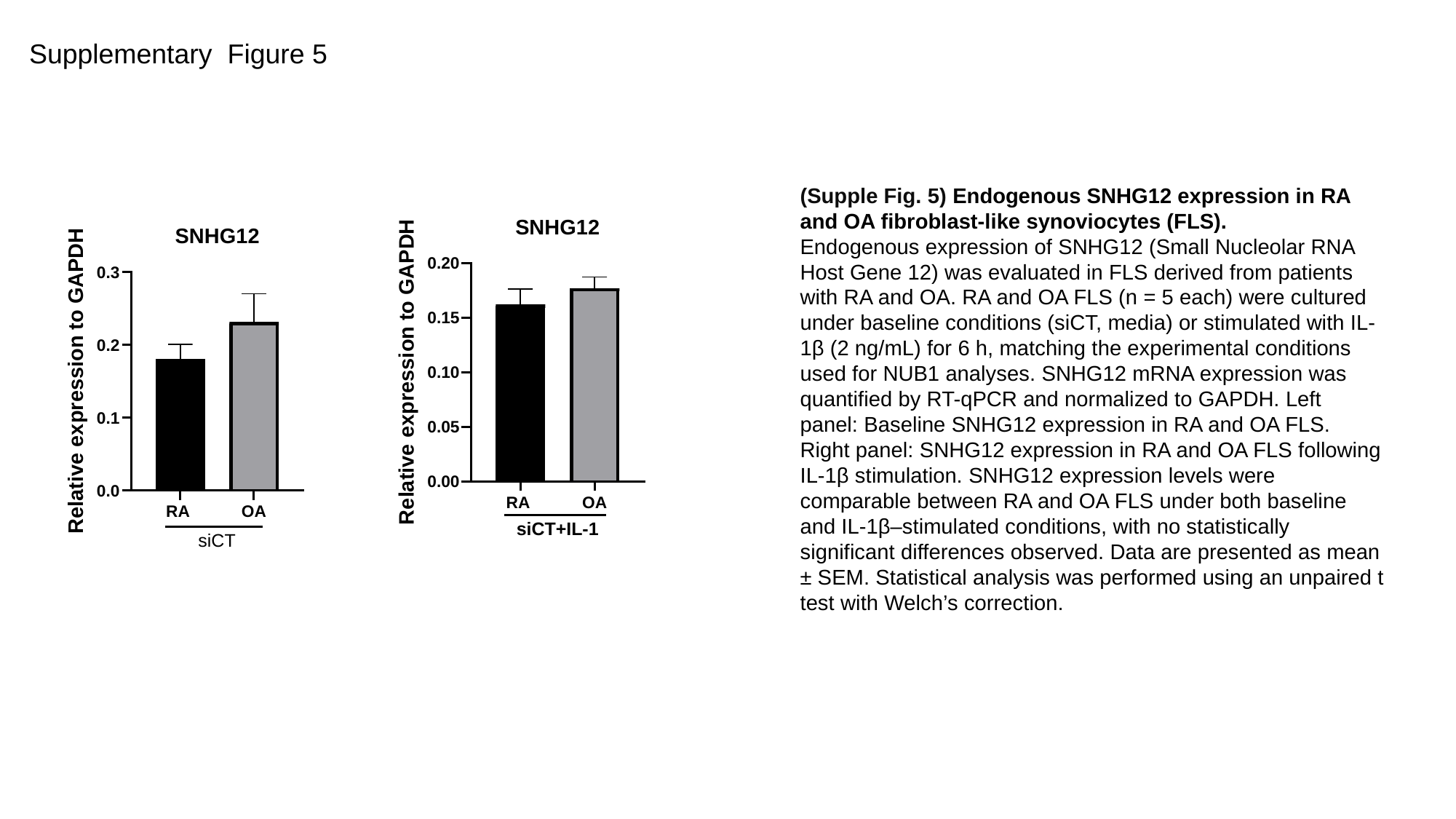

Supplementary Figure 5
(Supple Fig. 5) Endogenous SNHG12 expression in RA and OA fibroblast-like synoviocytes (FLS).
Endogenous expression of SNHG12 (Small Nucleolar RNA Host Gene 12) was evaluated in FLS derived from patients with RA and OA. RA and OA FLS (n = 5 each) were cultured under baseline conditions (siCT, media) or stimulated with IL-1β (2 ng/mL) for 6 h, matching the experimental conditions used for NUB1 analyses. SNHG12 mRNA expression was quantified by RT-qPCR and normalized to GAPDH. Left panel: Baseline SNHG12 expression in RA and OA FLS. Right panel: SNHG12 expression in RA and OA FLS following IL-1β stimulation. SNHG12 expression levels were comparable between RA and OA FLS under both baseline and IL-1β–stimulated conditions, with no statistically significant differences observed. Data are presented as mean ± SEM. Statistical analysis was performed using an unpaired t test with Welch’s correction.

## Slide 6
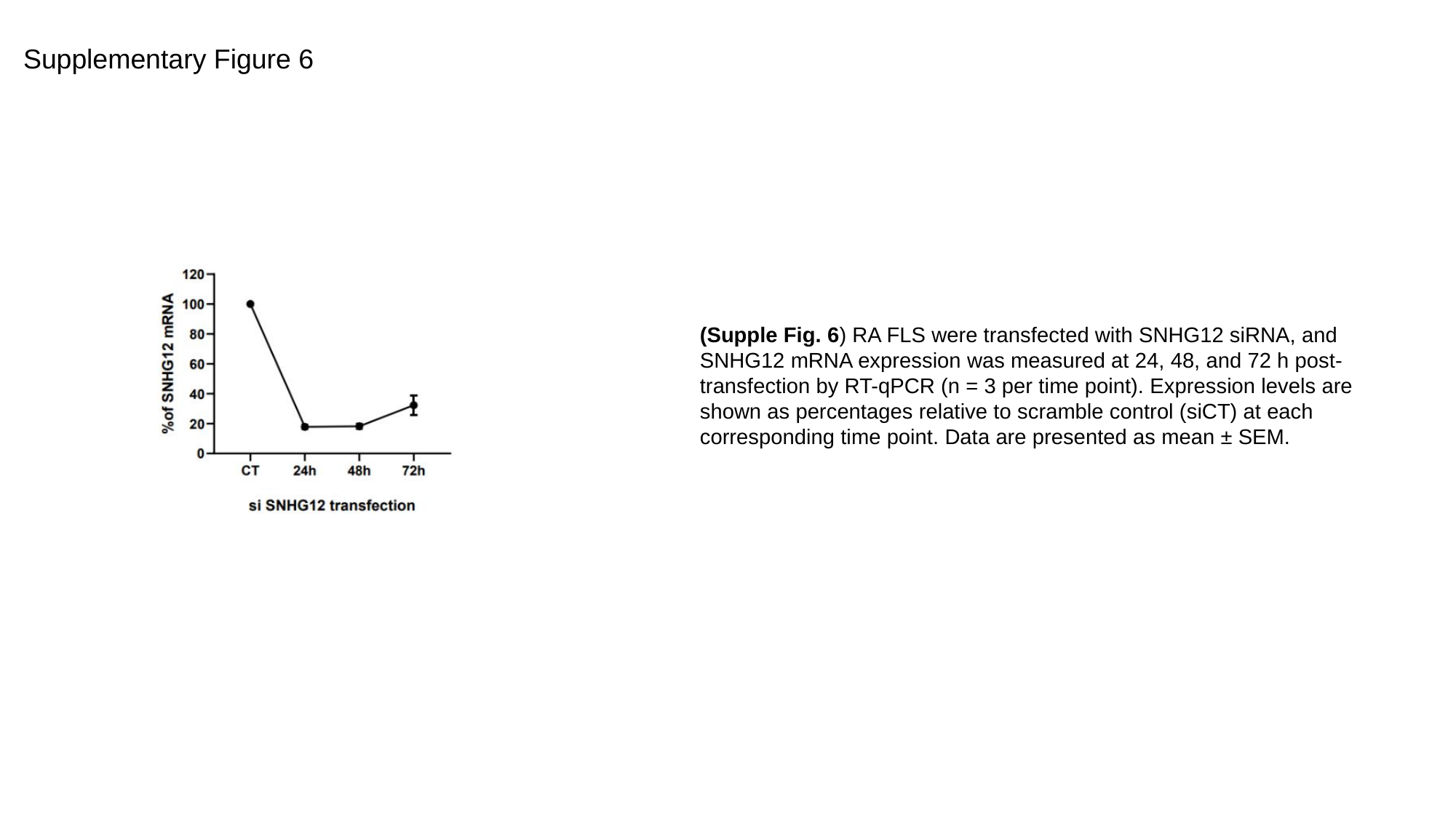

Supplementary Figure 6
(Supple Fig. 6) RA FLS were transfected with SNHG12 siRNA, and SNHG12 mRNA expression was measured at 24, 48, and 72 h post-transfection by RT-qPCR (n = 3 per time point). Expression levels are shown as percentages relative to scramble control (siCT) at each corresponding time point. Data are presented as mean ± SEM.

## Slide 7
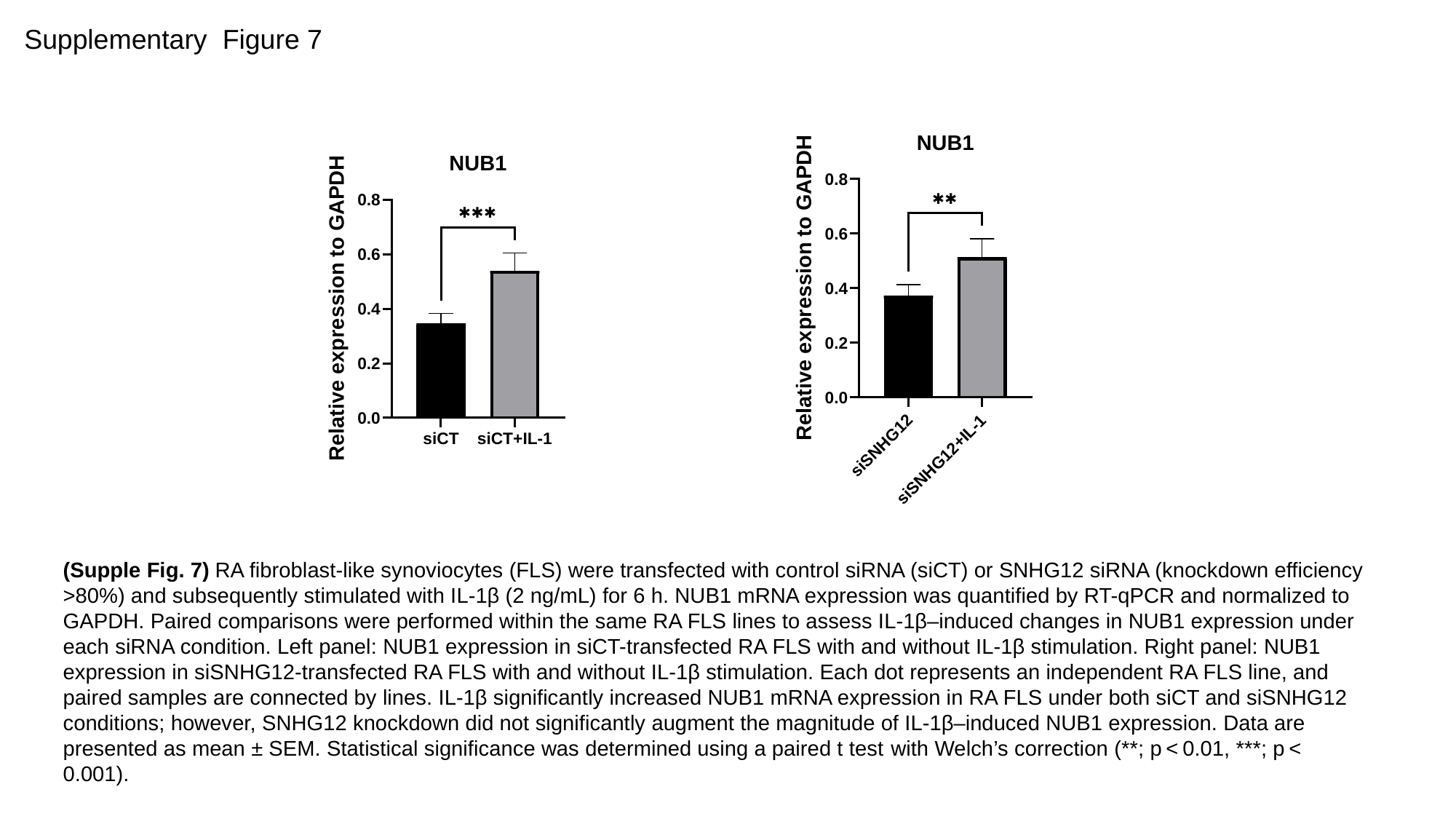

Supplementary Figure 7
(Supple Fig. 7) RA fibroblast-like synoviocytes (FLS) were transfected with control siRNA (siCT) or SNHG12 siRNA (knockdown efficiency >80%) and subsequently stimulated with IL-1β (2 ng/mL) for 6 h. NUB1 mRNA expression was quantified by RT-qPCR and normalized to GAPDH. Paired comparisons were performed within the same RA FLS lines to assess IL-1β–induced changes in NUB1 expression under each siRNA condition. Left panel: NUB1 expression in siCT-transfected RA FLS with and without IL-1β stimulation. Right panel: NUB1 expression in siSNHG12-transfected RA FLS with and without IL-1β stimulation. Each dot represents an independent RA FLS line, and paired samples are connected by lines. IL-1β significantly increased NUB1 mRNA expression in RA FLS under both siCT and siSNHG12 conditions; however, SNHG12 knockdown did not significantly augment the magnitude of IL-1β–induced NUB1 expression. Data are presented as mean ± SEM. Statistical significance was determined using a paired t test with Welch’s correction (**; p < 0.01, ***; p < 0.001).

## Slide 8
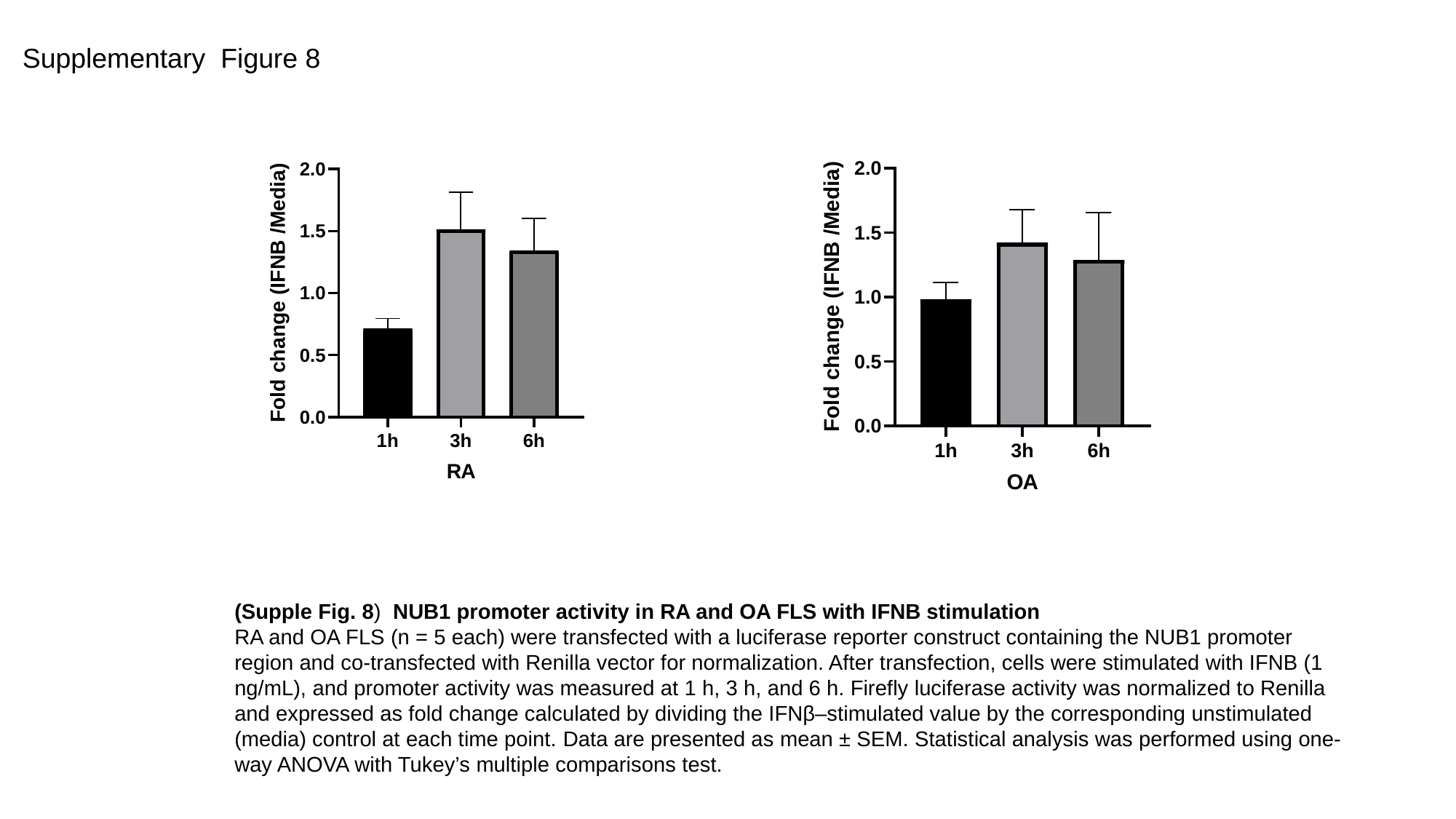

Supplementary Figure 8
(Supple Fig. 8) NUB1 promoter activity in RA and OA FLS with IFNB stimulation
RA and OA FLS (n = 5 each) were transfected with a luciferase reporter construct containing the NUB1 promoter region and co-transfected with Renilla vector for normalization. After transfection, cells were stimulated with IFNB (1 ng/mL), and promoter activity was measured at 1 h, 3 h, and 6 h. Firefly luciferase activity was normalized to Renilla and expressed as fold change calculated by dividing the IFNβ–stimulated value by the corresponding unstimulated (media) control at each time point. Data are presented as mean ± SEM. Statistical analysis was performed using one-way ANOVA with Tukey’s multiple comparisons test.

## Slide 9
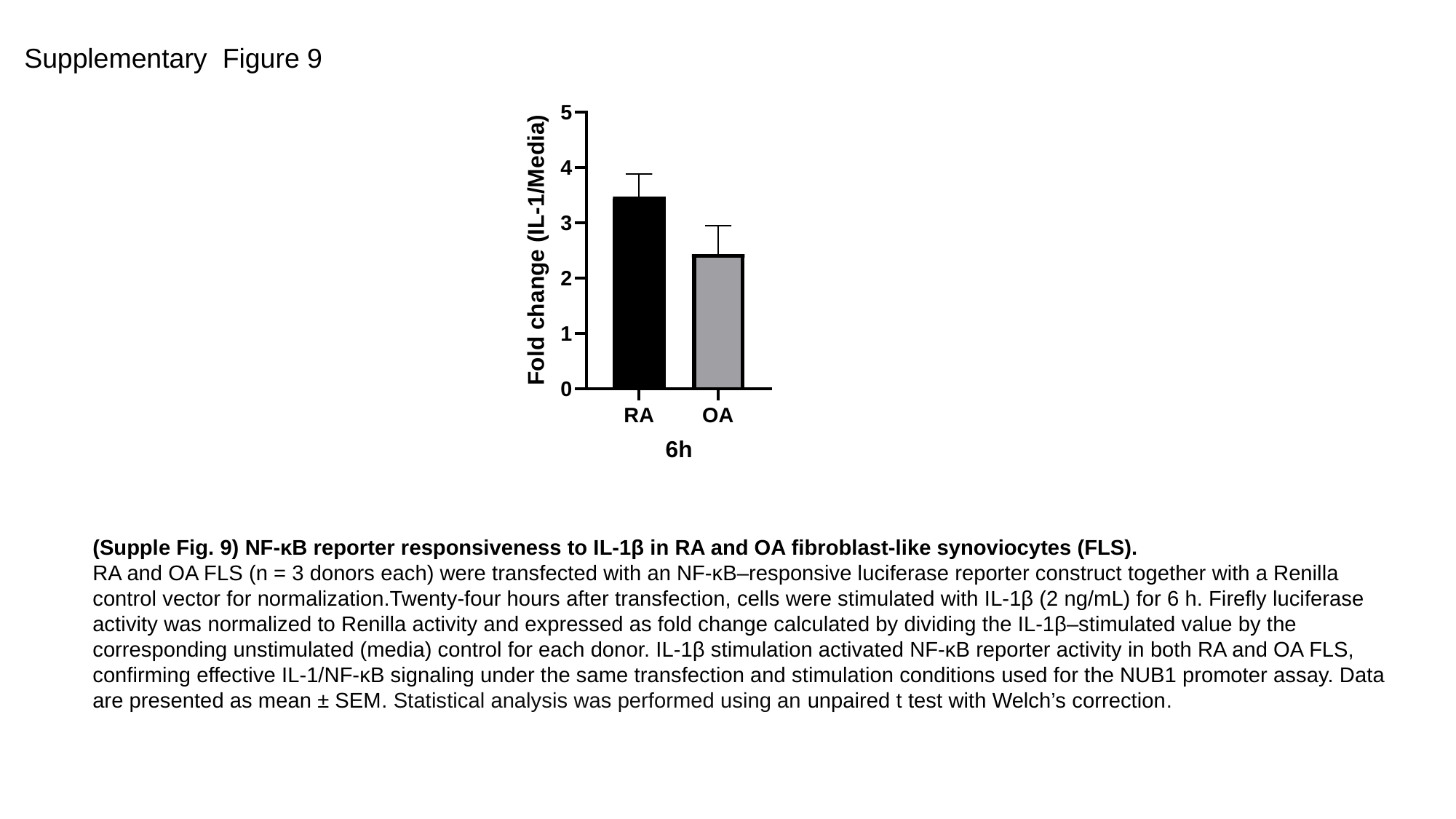

Supplementary Figure 9
(Supple Fig. 9) NF-κB reporter responsiveness to IL-1β in RA and OA fibroblast-like synoviocytes (FLS).
RA and OA FLS (n = 3 donors each) were transfected with an NF-κB–responsive luciferase reporter construct together with a Renilla control vector for normalization.Twenty-four hours after transfection, cells were stimulated with IL-1β (2 ng/mL) for 6 h. Firefly luciferase activity was normalized to Renilla activity and expressed as fold change calculated by dividing the IL-1β–stimulated value by the corresponding unstimulated (media) control for each donor. IL-1β stimulation activated NF-κB reporter activity in both RA and OA FLS, confirming effective IL-1/NF-κB signaling under the same transfection and stimulation conditions used for the NUB1 promoter assay. Data are presented as mean ± SEM. Statistical analysis was performed using an unpaired t test with Welch’s correction.

## Slide 10
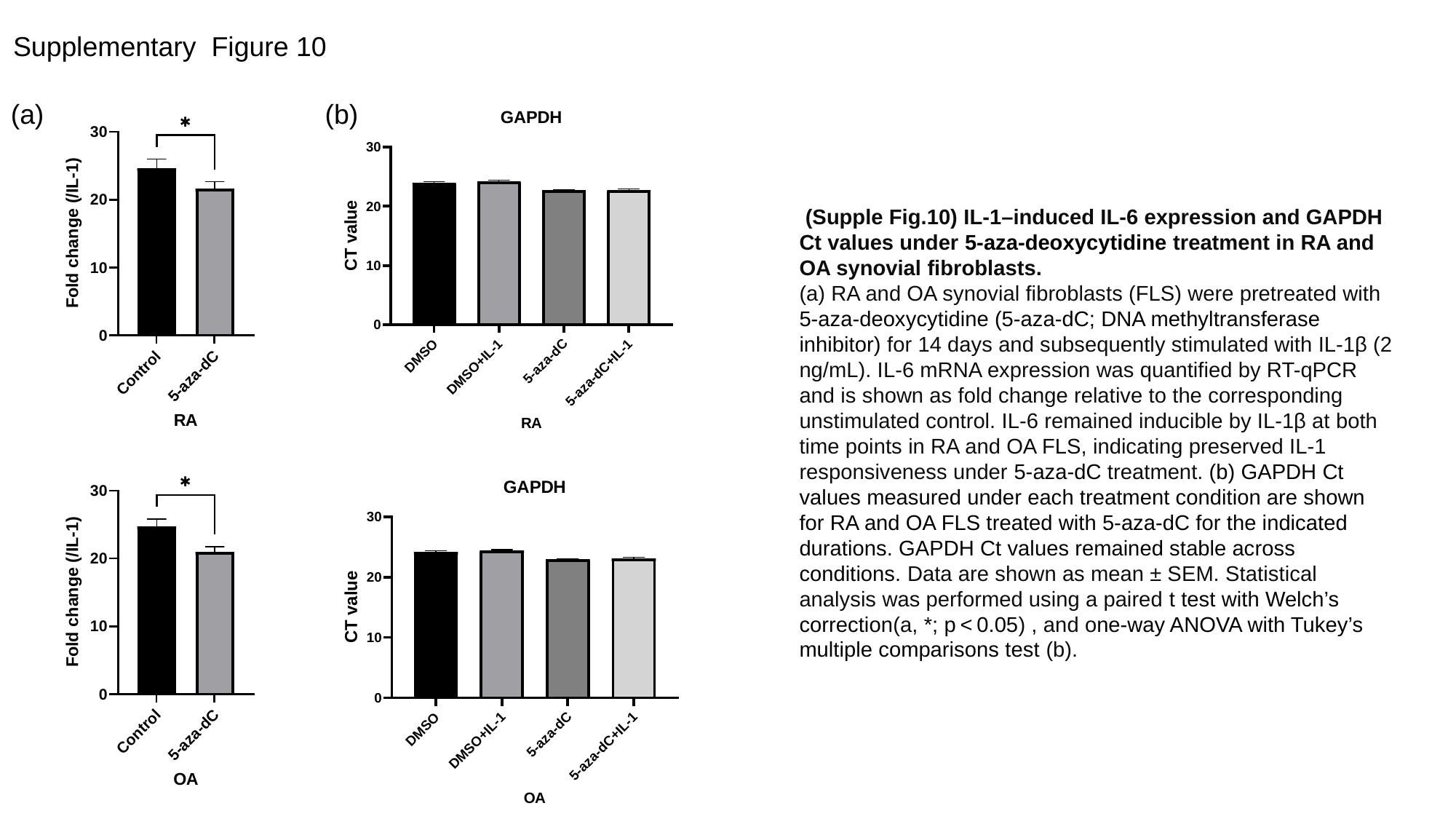

Supplementary Figure 10
(a)
(b)
 (Supple Fig.10) IL-1–induced IL-6 expression and GAPDH Ct values under 5-aza-deoxycytidine treatment in RA and OA synovial fibroblasts.
(a) RA and OA synovial fibroblasts (FLS) were pretreated with 5-aza-deoxycytidine (5-aza-dC; DNA methyltransferase inhibitor) for 14 days and subsequently stimulated with IL-1β (2 ng/mL). IL-6 mRNA expression was quantified by RT-qPCR and is shown as fold change relative to the corresponding unstimulated control. IL-6 remained inducible by IL-1β at both time points in RA and OA FLS, indicating preserved IL-1 responsiveness under 5-aza-dC treatment. (b) GAPDH Ct values measured under each treatment condition are shown for RA and OA FLS treated with 5-aza-dC for the indicated durations. GAPDH Ct values remained stable across conditions. Data are shown as mean ± SEM. Statistical analysis was performed using a paired t test with Welch’s correction(a, *; p < 0.05) , and one-way ANOVA with Tukey’s multiple comparisons test (b).

## Slide 11
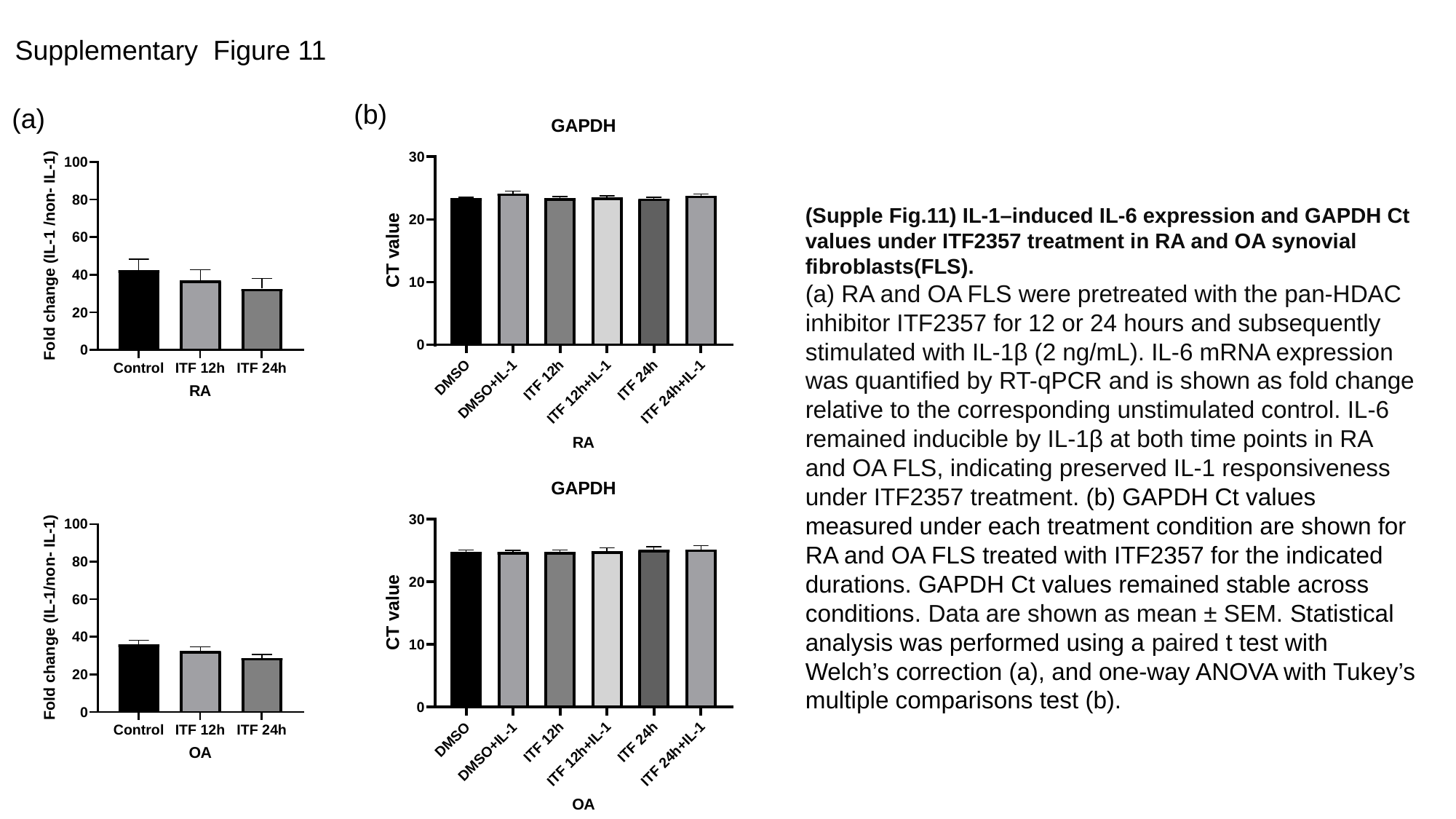

Supplementary Figure 11
(b)
(a)
(Supple Fig.11) IL-1–induced IL-6 expression and GAPDH Ct values under ITF2357 treatment in RA and OA synovial fibroblasts(FLS).
(a) RA and OA FLS were pretreated with the pan-HDAC inhibitor ITF2357 for 12 or 24 hours and subsequently stimulated with IL-1β (2 ng/mL). IL-6 mRNA expression was quantified by RT-qPCR and is shown as fold change relative to the corresponding unstimulated control. IL-6 remained inducible by IL-1β at both time points in RA and OA FLS, indicating preserved IL-1 responsiveness under ITF2357 treatment. (b) GAPDH Ct values measured under each treatment condition are shown for RA and OA FLS treated with ITF2357 for the indicated durations. GAPDH Ct values remained stable across conditions. Data are shown as mean ± SEM. Statistical analysis was performed using a paired t test with Welch’s correction (a), and one-way ANOVA with Tukey’s multiple comparisons test (b).

## Slide 12
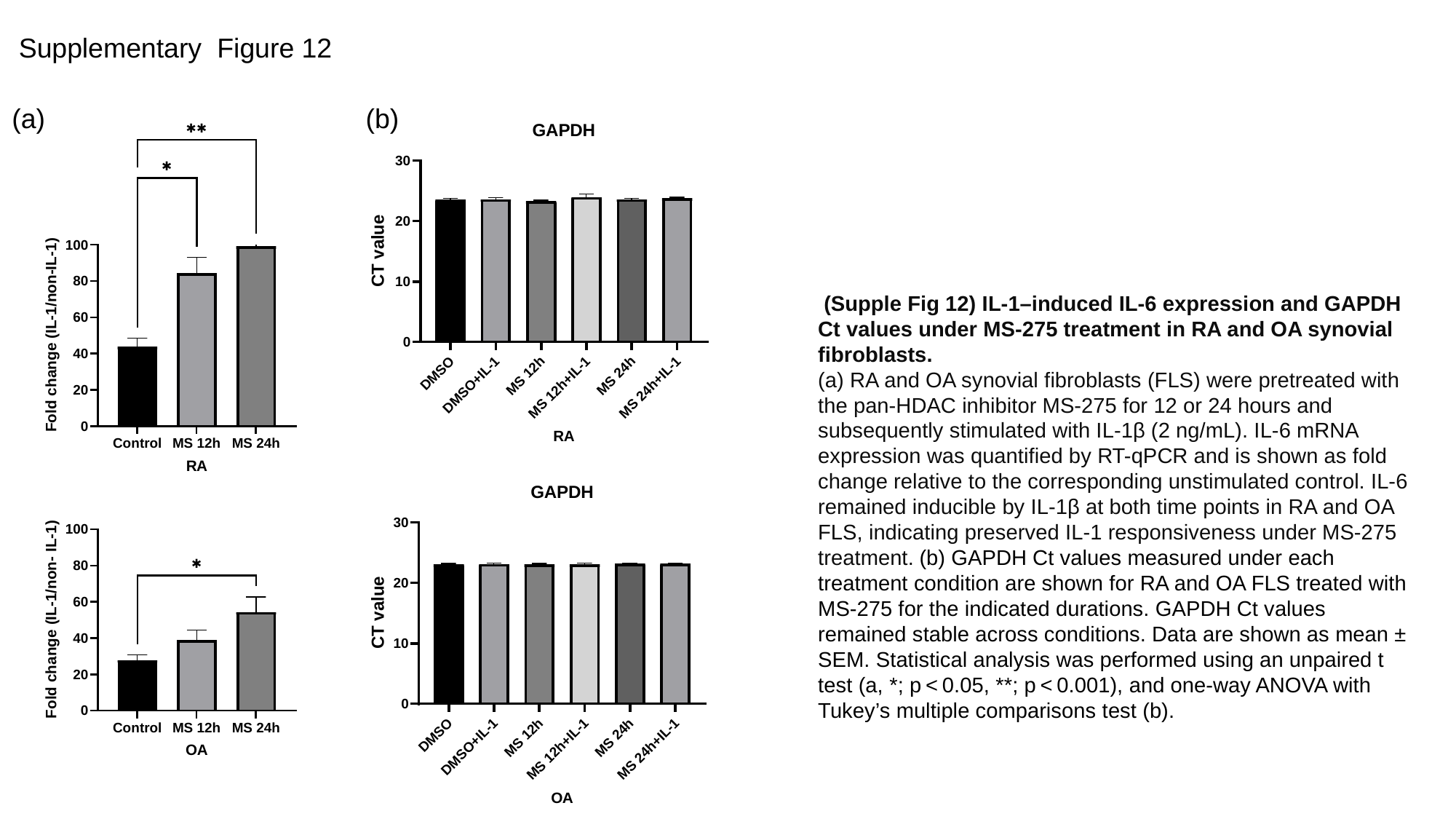

Supplementary Figure 12
(a)
(b)
 (Supple Fig 12) IL-1–induced IL-6 expression and GAPDH Ct values under MS-275 treatment in RA and OA synovial fibroblasts.
(a) RA and OA synovial fibroblasts (FLS) were pretreated with the pan-HDAC inhibitor MS-275 for 12 or 24 hours and subsequently stimulated with IL-1β (2 ng/mL). IL-6 mRNA expression was quantified by RT-qPCR and is shown as fold change relative to the corresponding unstimulated control. IL-6 remained inducible by IL-1β at both time points in RA and OA FLS, indicating preserved IL-1 responsiveness under MS-275 treatment. (b) GAPDH Ct values measured under each treatment condition are shown for RA and OA FLS treated with MS-275 for the indicated durations. GAPDH Ct values remained stable across conditions. Data are shown as mean ± SEM. Statistical analysis was performed using an unpaired t test (a, *; p < 0.05, **; p < 0.001), and one-way ANOVA with Tukey’s multiple comparisons test (b).
